# Structural Pharmacology of Bufotenine Derivatives in Activating the 5-HT_1A_ Receptor for Therapeutic Potential in Depression and Anxiety

**DOI:** 10.34133/research.0987

**Published:** 2025-12-23

**Authors:** Shu-jie Li, Qing-ning Yuan, Wen-yuan Wu, Zhi-han Chen, Duo Chen, Hong Shan, Qin-yu Chu, Wen Hu, Kai Wu, Tao Liu, Yu-yu Zhu, Li Hou, Jing Zhou, Jia Duan, Jin-ao Duan, H. Eric Xu, Hong-yue Ma

**Affiliations:** ^1^ Jiangsu Collaborative Innovation Center of Chinese Medicinal Resources Industrialization and Jiangsu Key Laboratory for High Technology Research of TCM Formulae, College of Pharmacy, Nanjing University of Chinese Medicine, Nanjing 210023, China.; ^2^ Department of Traditional Chinese Medicine, Fujian Medical University Union Hospital, Fuzhou 350000, Fujian, China.; ^3^ State Key Laboratory of Drug Research, Shanghai Institute of Materia Medica, Chinese Academy of Sciences, Shanghai 201203, China.; ^4^ School of Pharmaceutical Science and Technology, Hangzhou Institute for Advanced Study, UCAS, Hangzhou 310024, China.

## Abstract

The 5-HT_1A_ receptor is a critical target in the treatment of depression and anxiety. Bufotenine derivatives, such as 5-methoxy-*N*,*N*-dimethyltryptamine (5-MeO-DMT), 5-hydroxy-*N*,*N*-dimethyltryptamine (5-OH-DMT), and 5-hydroxy-*N*,*N*,*N*-dimethyltryptamine—derived from traditional Chinese medicine—have shown antidepressant potential. However, the structural basis of their interaction with 5-HT_1A_ and their pharmacological profiles remain incompletely understood. This study investigated bufotenine derivatives acting on multiple serotonin receptors, highlighting 5-HT_1A_ as a key mediator of antidepressant effects while recognizing 5-HT_2A_ as primarily responsible for hallucinogenic outcomes, to identify candidates with therapeutic efficacy but reduced hallucinogenic liability. We determined the cryo-electron microscopy structures of 5-HT_1A_ bound to selected bufotenine derivatives. Functional assays in mice, including behavioral tests and receptor activation studies, were used to evaluate the antidepressant of each compound. Structural analysis revealed that all bufotenine derivatives engage conserved motifs within the 5-HT_1A_ binding pocket, with 5-OH-DMT displaying a distinct interaction pattern. Behavioral assays showed that 5-OH-DMT and 5-MeO-DMT retained strong antidepressant and anxiolytic effects. These pharmacological differences correlate with their unique receptor binding conformation. This study delineated the structural pharmacology of bufotenine derivatives at the 5-HT_1A_ receptor, identifying 5-OH-DMT and 5-MeO-DMT as promising antidepressant and anxiolytic candidates. The findings establish a molecular framework for the development of next-generation nonhallucinogenic therapeutics aimed at 5-HT_1A_.

## Introduction

Depression is a prevalent mental illness that affects the quality of life and psychosocial performance. It causes depression, anhedonia, poor self-esteem, guilt, worthlessness, weariness, sleep problems, and suicidal thoughts [1,2]. Standard antidepressants like monoamine oxidase inhibitors and selective serotonin reuptake inhibitors regulate central nervous system monoamine neurotransmitter levels. The therapeutic effects of these drugs usually take weeks to appear [3]. About two-thirds of patients relapse or do not respond to these treatments [4,5]. The monoamine hypothesis, which states that neurotransmitter imbalances in serotonin (5-HT), norepinephrine, and dopamine cause depression, underpins the efficacy and pharmacodynamics of first- and second-generation antidepressants [6,7]. Depressive disorders may be caused by at least 6 serotonin receptor subtypes (5-HT_1A_, 5-HT_1B_, 5-HT_1D_, 5-HT_2A_, 5-HT_2C_, and 5-HT_7_), according to emerging evidence [8,9].

Serotonin dysregulation is largely considered a key cause of severe depressive illness. The neuropathology of depression depends on the 5-HT_1A_ receptor (5-HT_1A_R), the main serotonin receptor [10,11]. 5-HT_1A_R regulates melancholy and anxiety [12]. In mice, 5-HT_1A_R-deficient animals exhibit increased anxiety, dread, and freezing, demonstrating its function in anxiety regulation [13]. In addition, overexpression of 5-HT_1A_R in the forebrain during early postnatal development, but not in the raphe nuclei, restores conventional behavioral features in knockout mice, underscoring the importance of postsynaptic 5-HT_1A_R in mental health [14]. Mice and primates with reduced 5-HT_1A_R expression exhibit anxiety and stress-related behaviors [15,16]. Both psychedelics and selective 5-HT_1A_R agonists exhibit anxiolytic properties through distinct yet partly overlapping mechanisms. Clinically used 5-HT_1A_ agonists such as buspirone alleviate anxiety by modulating serotonergic signaling and stress-coping pathways. Psychedelics like psilocybin, which also engage 5-HT_1A_Rs alongside 5-HT_2A_ activation, demonstrate additional anxiolytic and antidepressant potential. 5-HT_1A_R is a target of licensed anxiolytic and antidepressant drugs including buspirone (BuSpar) [17] and vilazodone (Viibryd) [18]; therefore, knowing its processes is crucial to improving depression therapies. Through Gi/o family heterotrimeric G proteins, 5-HT_1A_R inhibits adenylyl cyclase and lowers cyclic adenosine monophosphate (cAMP) levels, and this effect is enhanced by 5-HT_1A_R agonists [19]. Despite extensive investigation, the molecular abnormalities in the serotonin pathway linked to clinical depression and suicidality are still unknown.

Traditional antidepressants have limited effectiveness, extensive treatment times, and lasting adverse effects, underscoring the need for innovative and fast-acting treatments [20–22]. Psychoactive bufotenine compounds from *Bufo alvarius* toad parotid glands affect humans and animals. Bufotenine, a natural tryptamine in Ch’an Su, has been utilized in Chinese medicine for centuries [23]. Bufotenine derivatives have an indole core, an amino group, and an ethyl side chain like serotonin. These derivatives include 5-methoxy-*N*,*N*-dimethyltryptamine (5-MeO-DMT), 5-hydroxy-*N*,*N*-dimethyltryptamine (5-OH-DMT), and 5-hydroxy-*N*,*N*,*N*-dimethyltryptamine (5-OH-TMT). Studies reveal that indole ring alterations at position 5 increase potency relative to that of other substituted and unsubstituted tryptamines [24], but clinical effects are similar [25]. Recent discoveries that hallucinogens may treat depression, anxiety, drug use disorders, and obsessive-compulsive behaviors have increased interest in their pharmacology [26,27]. Some tryptamines, both 5-MeO-substituted and unsubstituted, block or release 5-HT [28]. This combined mechanism of 5-HT reuptake inhibition and receptor interaction may be better than selective serotonin reuptake inhibitors for mood disorders [29]. Most structure–activity relationship research on tryptamines has concentrated on indole ring replacements, with few investigations on terminal amino group modifications [30]. Some 5-MeO-tryptamines bind with the 5-HT_2A_ receptor (5-HT_2A_R), although they usually work better at 5-HT_1A_R [31,32]. However, inconsistent results have been reported [28]. Additionally, bufotenine derivatives’ 5-HT_1A_R modulation effect in depression therapy is unknown.

Extensive research has established that psychedelics function as 5-HT_2A_R agonists, capable of inducing profound alterations in perception, cognition, and mood states [33]. However, emerging evidence suggests that the therapeutic efficacy of 5-HT_2A_R agonists may not necessarily depend on psychedelic experiences, with studies demonstrating that psilocybin’s antidepressant effects can occur independently of both 5-HT_2A_R activation and associated psychedelic activity [9,34,35]. These findings highlight a critical knowledge gap regarding the mechanistic relationship between psychedelic properties and therapeutic outcomes in 5-HT_2A_R-targeted interventions. The clinical translation of psychedelic-based therapies faces substantial methodological and practical challenges. The compounds’ intrinsic psychoactive properties fundamentally undermine the integrity of double-blind clinical trial designs, while the standard practice of combining pharmacological intervention with psychotherapy obscures the precise attribution of therapeutic benefits. Additional concerns regarding long-term safety profiles and potential for nonmedical use further complicate their clinical implementation. Motivated by increasing scientific interest in microdosing protocols for cognitive enhancement and mood regulation [36], we sought to develop a novel therapeutic agent that maintains robust antidepressant efficacy while exhibiting minimized psychedelic properties, thereby addressing current limitations in the field.

This study explored the structural and functional mechanisms of how bufotenine derivatives, namely, 5-MeO-DMT, 5-OH-DMT, and 5-OH-TMT, bind to and activate 5-HT_1A_R at molecular and atomic levels. Furthermore, we systematically investigated the therapeutic potential of bufotenine derivatives in established mouse models of depression. Our study shows that 5-OH-DMT acts on multiple serotonin receptors, with 5-HT_1A_ contributing to its antidepressant and anxiolytic effects and reduced 5-HT_2A_ involvement accounting for diminished hallucinogenic activity. These results provide a pharmacological foundation for the development of next-generation antidepressant therapies with targeted 5-HT_1A_R engagement.

## Results

### Bufotenine derivatives acting at 6 depression-related serotonin receptors

**Fig. 1.**
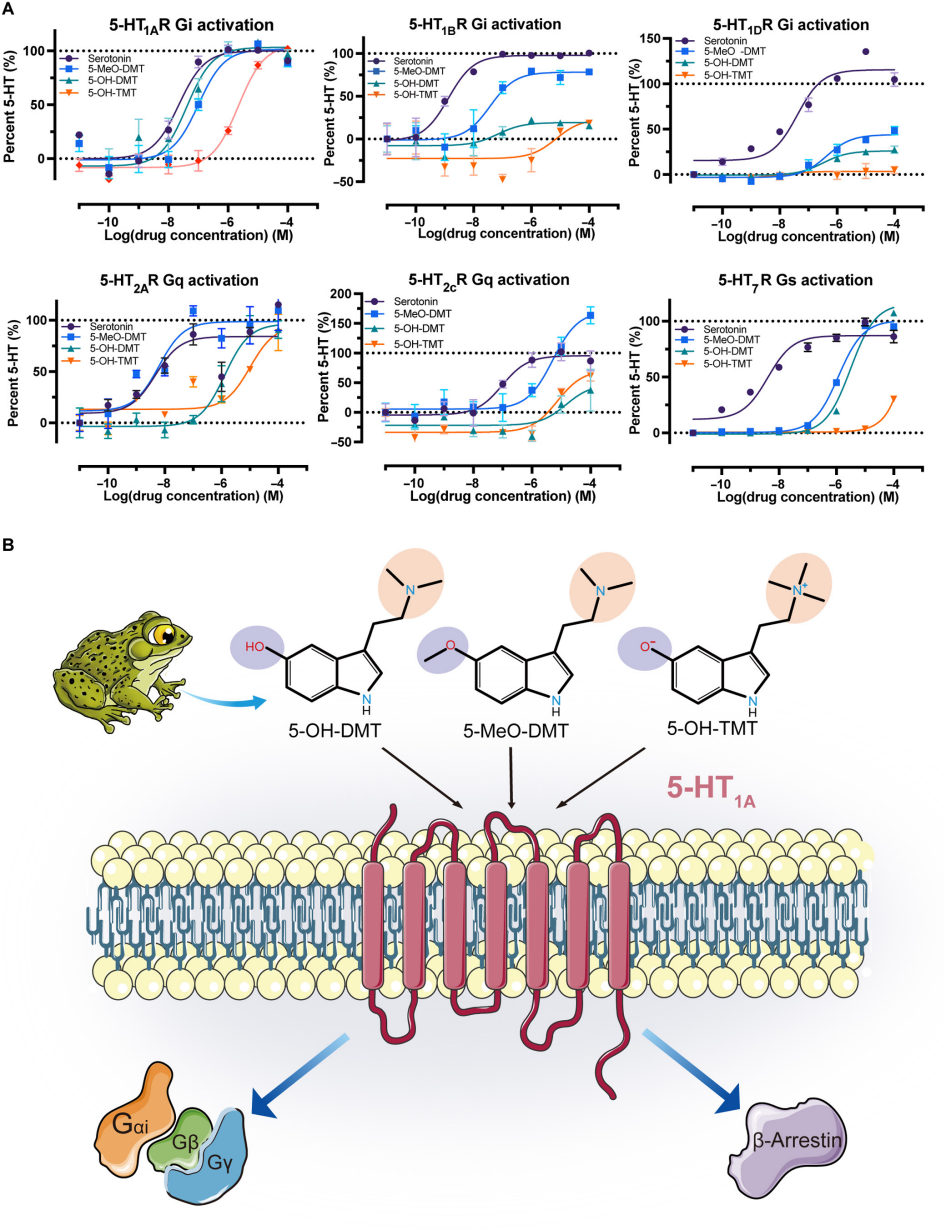
Bufotenine derivatives acting at 6 depression-related serotonin receptors. (A) The difference of bufotenine derivative in activating depression-related serotonin receptors. (B) Schematic illustration of G-protein coupling of 5-HT_1A_ activated by a bufotenine derivative. 5-HT, serotonin; 5-HT_1A_R, 5-HT_1A_ receptor; 5-HT_1B_R, 5-HT_1B_ receptor; 5-HT_1D_R, 5-HT_1D_ receptor; 5-HT_2A_R, 5-HT_2A_ receptor; 5-HT_2C_R, 5-HT_2C_ receptor; 5-HT_7_R, 5-HT_7_ receptor; 5-MeO-DMT, 5-methoxy-*N*,*N*-dimethyltryptamine; 5-OH-DMT, 5-hydroxy-*N*,*N*-dimethyltryptamine; 5-OH-TMT, 5-hydroxy-*N*,*N*,*N*-dimethyltryptamine.

We evaluated the impact of bufotenine derivatives on 6 serotonin receptor subtypes associated with depression (5-HT_1A_, 5-HT_1B_, 5-HT_1D_, 5-HT_2A_, 5-HT_2C_, and 5-HT_7_) using the GloSensor cAMP assay to measure Gi and Gs activation and the IP-One assay to assess Gq activation, thereby detecting signaling activity at each receptor. All synthesized bufotenine derivatives demonstrated potency at 5-HT receptor subtypes, with particularly pronounced potency and efficacy observed at 5-HT_1A_Rs (Fig. 1A). 5-OH-DMT demonstrated the greatest potency for 5-HT_1A_R with an EC_50_ of 0.1 μM. The activation potency at 5-HT_1A_R was lower for 5-MeO-DMT (EC_50_ = 0.5 μM) and 5-OH-TMT (EC_50_ = 10 μM) compared to those for other tryptamines. We conducted a thorough pharmacological analysis of bufotenine derivatives to evaluate their hallucinogenic potential via 5-HT_2A_R activation, comparing their receptor binding profiles and functional activities at 5-HT_1A_R and 5-HT_2A_R subtypes. 5-MeO-DMT demonstrates markedly greater potency at 5-HT_2A_R (EC_50_ = 0.01 μM) compared to 5-OH-DMT (EC_50_ = 1 μM), being 100 times more potent. 5-OH-TMT showed a similar attraction to 5-HT_1A_Rs (EC_50_ = 10 μM). The efficacy of 5-HT_1A_R was greater for these compounds compared to that of 5-HT_2A_R. Bufotenine derivatives generally showed greater potency for 5-HT_1A_R than for 5-HT_2A_R. The functional profile of 5-MeO-DMT closely aligns with the recently reported structural findings [37], with an approximately 10-fold difference in EC_50_ values, a variation that remains within an acceptable and interpretable range. In our experiments, bufotenine derivatives acted as full agonists in 5-HT_1A_-mediated G-protein signaling compared to serotonin, despite variations in selectivity and potency. These findings validate the potency and efficacy of 5-MeO-DMT at 5-HT_1A_, corroborating previous research highlighting the importance of 5-HT_1A_ in vivo pharmacology [38,39]. Our research demonstrates that 5-OH-DMT exhibits greater efficacy as a 5-HT_1A_ agonist compared to 5-MeO-DMT and 5-OH-TMT, with 5-OH-TMT being the least potent.

### Structures of bufotenine-derivative-bound 5-HT_1A_

**Fig. 2.**
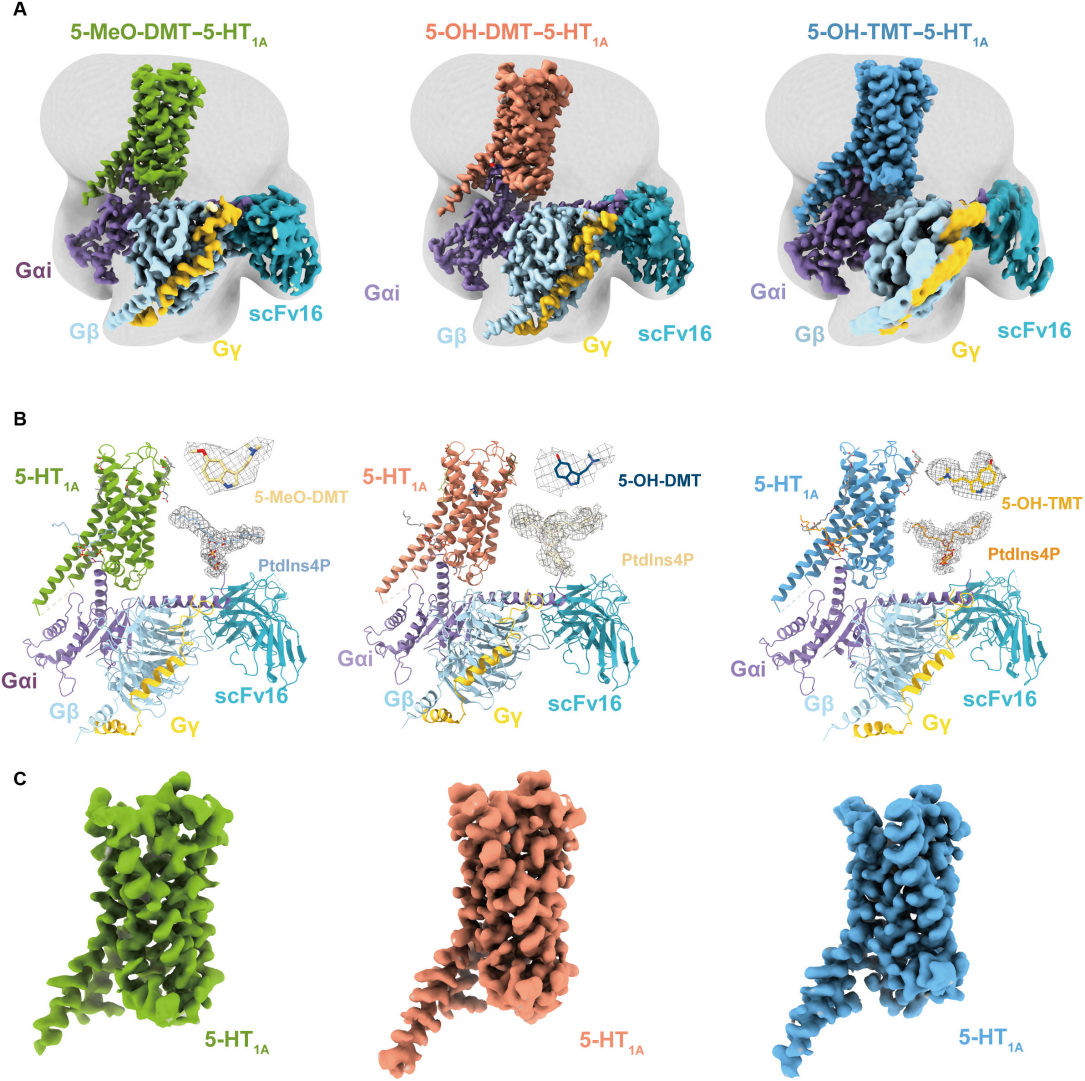
Cryo-electron microscopy (cryo-EM) structures of 5-MeO-DMT–5-HT_1A_–Gi–scFv16, 5-OH-DMT–5-HT_1A_–Gi–scFv16, and 5-OH-TMT–5-HT_1A_–Gi–scFv16 complexes. (A to C) Cryo-EM density maps (A and C) and cartoon representation (B) of 5-MeO-DMT–5-HT_1A_–Gi–scFv16, 5-OH-DMT–5-HT_1A_–Gi–scFv16, and 5-OH-TMT–5-HT_1A_–Gi–scFv16. Composite DeepEMhancer sharpened cryo-EM density maps colored by protein components. PtdIns4P, phosphatidylinositol-4-phosphate.

We generated 5-HT receptor–Gi complexes by coexpressing the receptors with a dominant-negative variant of human Gαi1 [40] and human Gβγ. The 5-HT_1A_–Gi complexes coupled to 5-OH-TMT, 5-MeO-DMT, or 5-OH-DMT were analyzed, and their structures were determined at a resolution of 2.6 to 2.9 Å. The 5-MeO-DMT-bound 5-HT_1A_–Gi complex and the 5-OH-DMT-bound HT_1A_–Gi complex structures were resolved at a 2.9-Å resolution. The cryo-electron microscopy (cryo-EM) maps offer sufficient clarity to accurately identify the positions of the receptor, G-protein trimer, and bound ligands within the receptor–G-protein complexes (Fig. 2A to C, Figs. S1 to S4, and Table S1). Our structure shows a high degree of similarity to the recently reported 5-MeO-DMT–5-HT_1A_ complex, with only minor differences (Fig. S6F) [37]. The cryo-EM maps of 5-HT_1A_ with 5-OH-TMT, 5-MeO-DMT, and 5-OH-DMT show unique structures that align precisely with a phosphatidylinositol-4-phosphate (PtdIns4P) molecule [41]. Our previous research demonstrated that PtdIns4P acts as a positive allosteric modulator of 5-HT_1A_ by enhancing its basal activity and facilitating Gi recruitment and that 5-HT_1A_ is further surrounded by diverse lipid species at the membrane interface [41].

### Binding mode of bufotenine derivatives at 5-HT_1A_

**Fig. 3.**
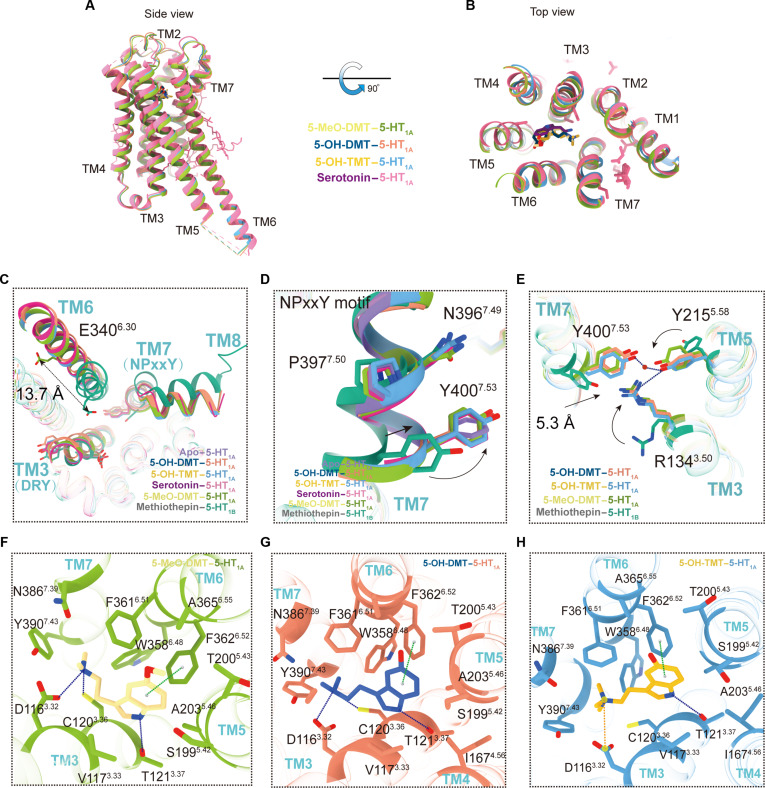
Structural features of 5-HT_1A_ in the active state by bufotenine derivatives. (A and B) Structural superposition of bufotenine-derivative-bound 5-HT_1A_ and 5-HT-bound 5-HT_1A_, presented from both the side view (left) and top view (right). (C to E) Conformational changes of the conserved “microswitches” upon receptor activation. The outward movement of the TM7 of the active receptor and the conformational changes of residue side chains are highlighted as black arrows. (F to H) Polar interaction network that stabilizes the conformation of bufotenine-derivative-bound 5-HT_1A_, respectively (dotted blue line, hydrogen bonds; ochre brown, salt bridges; dotted green line, π–π interactions).

The structures we have discovered demonstrate distinct interactions between drugs and receptors within the orthosteric binding pocket (OBP), perhaps resulting in variations in pharmacological effects. The binding position of bufotenine derivatives in 5-HT_1A_R complexes closely resembles that of serotonin within the receptor’s structure (Fig. 3A and B). Bufotenine derivatives binding to the orthosteric pocket of 5-HT_1A_ complexes apply downward pressure on the toggle switch residue W358^6.48^ (Fig. S5A), leading to conformational changes in F354^6.44^ and I124^3.40^ within the PIF motif (Fig. S5B) and R134^3.50^ in the DRY motif (Fig. S5C). Conformational changes disrupt the conserved ionic lock between TM3 and TM6, leading to a notable outward shift of TM6 by approximately 13.7 Å at the Cα of E340^6.30^ at the cytoplasmic end of TM6 (Fig. 3C). The inward shift of approximately 5.3 Å at the cytoplasmic end of TM7 enables hydrogen bonding between the Y400^7.53^ of the NPxxY motif and Y215^5.58^ with the R134^3.50^ of the DRY motif (Fig. 3D and E). These conformational alterations facilitate the accessibility of the receptor’s cytoplasmic pocket for Gi binding.

The identification of bufotenine derivatives relies on 2 key chemical elements: the indole ring and the ethylamine side chain (Fig. S6A to C), which mostly occupy the OBP. The indole ring serves as the fundamental structure of bufotenine derivatives. The indole ring primarily participates in π–π interactions with F361^6.51^ and F362^6.52^, as Ballesteros–Weinstein numbering (Fig. 3F to H) [42]. In the lower region of the 5-HT_1A_ binding pocket, the indole nitrogen of bufotenine derivatives establishes a hydrogen bond with T121^3.37^ (Fig. 3F to H), causing a 16.0° to 39.3° angle rotation of the bufotenine derivatives around the axis of the indole nitrogen (Fig. S6D).

Bufotenine derivatives are primarily distinguished by the position of the indole ring, specifically at the 3 and 5 locations, with an ethylamine side chain attached to the 3 position (Fig. S6A to C). This side chain imparts tryptamine characteristics to the bufotenine derivatives. The indole ring has a hydroxyl group at the 5-position. This property also differentiates the bufotenine derivatives from one another (Fig. S6A to C). Furthermore, the indole ring of 5-OH-DMT establishes a hydrophobic contact with I189^ECL2^ located in extracellular loop 2 of 5-HT_1A_R (Fig. S6E). This is a notable distinction among the 3 derivatives that is not observed in the other 2 compounds. Both 5-OH-DMT and 5-OH-TMT were observed to have the indole ring interacting with I167^4.56^; however, this interaction was not observed in 5-MeO-DMT (Fig. 3F to H). Additionally, the indole ring forms notable interactions with adjacent residues within the pocket, including V117^3.33^, S199^5.42^, T200^5.43^, A203^5.46^, and A365^6.51^ (Fig. 3F to H and Fig. S7). The ethylamine side chain has the ability to establish a hydrogen bond with the nearby D116^3.32^, which results in a certain alignment of the bufotenine derivatives (Fig. 3F to H). The positive charge on the ethylamine side chain of 5-OH-TMT prevents additional hydrogen bonding with adjacent nitrogen atoms. Nevertheless, this favorable charge also promotes the stability of the ligand within the pocket by salt bridge interactions (Fig. 3H). The ethylamine side chain interacts with the receptor via residues C120^3.36^, W358^6.48^, N386^7.39^, and Y390^7.43^ (Fig. S6E). In general, the alterations made to the ligand do not change the way it binds. Similar to the binding mechanism of serotonin, we discovered that the sites D116^3.32^ and T121^3.37^ were crucial in the binding of bufotenine derivatives to 5-HT_1A_ (Fig. S6E). Following the mutation of the D116^3.32^ and T121^3.37^ sites to alanine, EC_50_ decreased by around 100-fold.

Through the alanine mutants of F361^6.51^ and W358^6.48^, we discovered a significant decrease in the potency of the 3 bufotenine derivatives in activating 5-HT_1A_R (Fig. S7 and Table S2). F361^6.51^ stabilizes the tryptamine scaffold’s structure and is essential for the efficacy of different amine replacements. Previous studies have shown that A361^6.51^, located adjacent to the 4-indole and 5-indole substituents in the 5-HT receptor, is specifically linked to 5-HT_1A_R and is essential for determining its ligand selectivity (Fig. 2G to I and Fig. S7B, E, and H). In order to examine the underlying mechanism of ligand selectivity, we introduced a mutation at position A365^6.55^, replacing it with phenylalanine. We detected a reduction in the potency of all analyzed tryptamine derivatives, with 5-OH-DMT exhibiting the most significant loss in potency, estimated to be around 1,000-fold less potent (Fig. S7E). Therefore, the presence of A365^6.55^ in 5-HT_1A_R is crucial for the strong effectiveness of 5-substituted tryptamines. This may be because A365^6.55^ has the capacity to accept groups with diverse chemical characteristics.

### Structural insights from bufotenine-derivative-bound 5-HT_2A_R

**Fig. 4.**
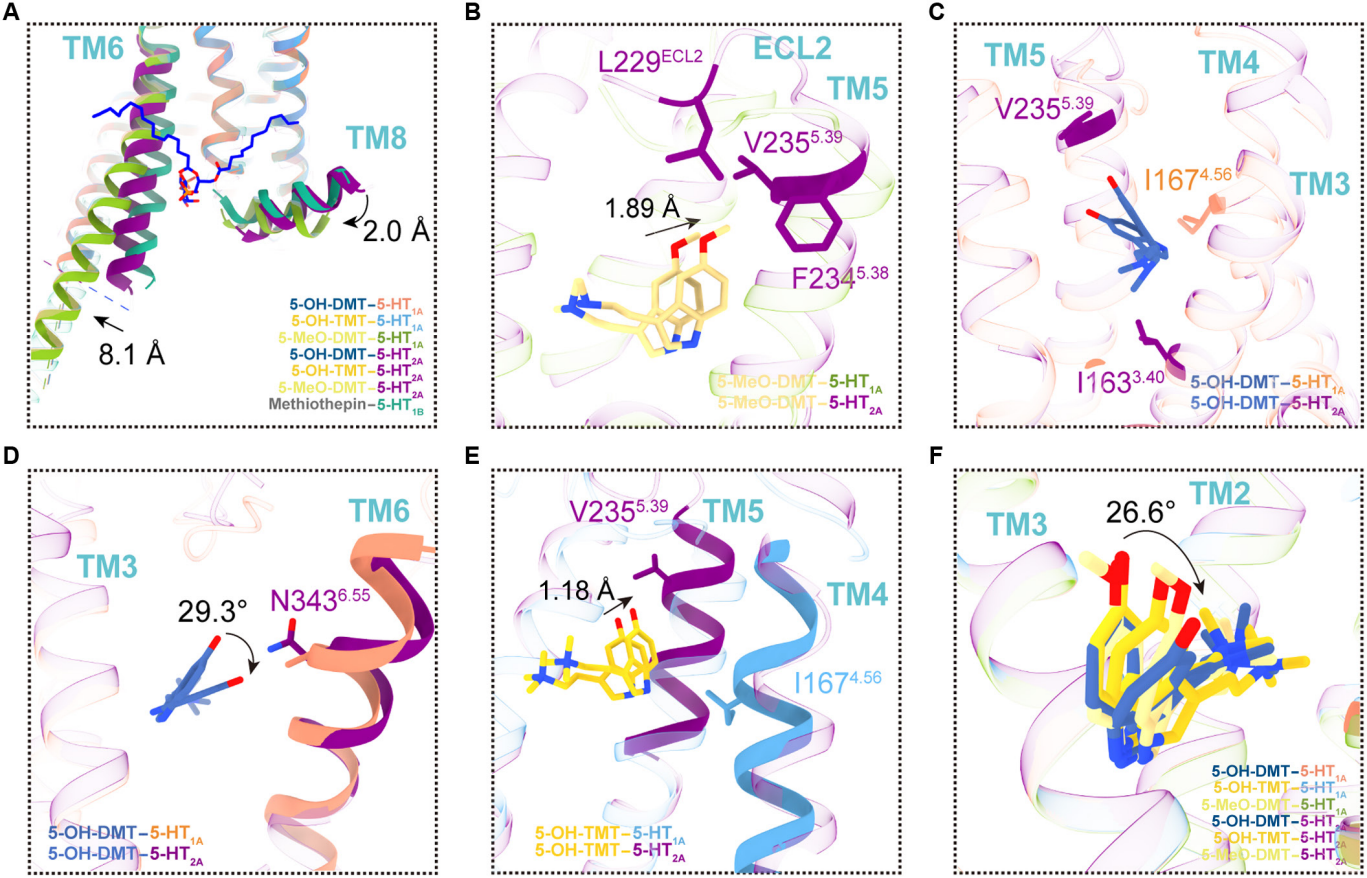
Structural comparison of bufotenine derivatives in relation to 5-HT_1A_R, 5-HT_1B_R, and 5-HT_2A_R. (A) 5-HT_1A_R displaces TM6 and TM8 outward and downward during activation, attributed to PtdIns4P binding between TM6 and TM7. Aligned structures of the inactive-state 5-HT_1B_ (bound to the inverse agonist methiothepin; PDB: 6G79). (B) Unlike the 5-MeO-DMT–5-HT_1A_ structure, 5-MeO-DMT–5-HT_2A_ shifts the ligand toward TM5 during activation. (C and D) The 5-OH-DMT–5-HT_1A_ structure experiences rotation of the indole ring during activation. (E) The ligand is shown to migrate toward TM5 in the 5-OH-TMT–5-HT_2A_ structure. (F) In contrast to 5-HT_2A_, the indole ring of bufotenine derivatives in 5-HT_1A_ is rotated, modifying the local polarity effect. The black arrows indicate the trajectory of the receptor helix or the residues’ side chain’s migration.

To investigate the variations in the strength of bufotenine derivatives at 5-HT_1A_R and 5-HT_2A_R, we employed molecular docking to forecast the arrangement of bufotenine derivatives binding with 5-HT_2A_ (PDB: 7RAN). In comparison to 5-HT_2A_R, 5-HT_1A_R exhibited an outward displacement of about 8.1 Å in the TM6 region, whereas TM8 was elevated by almost 2 Å (Fig. 4A). This change may be associated with the binding of PtdIns4P between TM6 and TM7. It is important to mention that 5-MeO-DMT has greater interactions with the V235^5.39^ and F234^5.38^ residues on TM5 in 5-HT_2A_. This, together with the influence of L229^ECL2^, causes a shift of about 1.89 Å toward TM5 (Fig. 4B). The structural analysis of 5-OH-DMT–5-HT_2A_ reveals that the I163^3.40^ residue on TM3 and the V235^5.39^ residue on TM5 interact with the ligand (Fig. 4C). Unfortunately, the I167^4.56^ residue site is absent on TM4. Furthermore, the indole ring of the ligand linked to 5-HT_2A_ undergoes a 25° upward rotation when N343^6.55^ replaces A365^6.55^ on 5-HT_1A_ TM6 (Fig. 4D). These circumstances may cause a significant drop in potency and reduced efficacy with 5-HT_2A_, roughly 10 times lower than normal (Fig. 1A). The occurrence of 5-OH-TMT in 5-HT_2A_ results in a displacement of about 1.18 Å toward TM5. This displacement might be attributed to the lack of I167^4.56^ on TM4 and the presence of V235^5.39^ on TM5 (Fig. 4E). Mutation data also demonstrated that 5-OH-TMT exhibited no alteration in affinity for both receptors but displayed a small increase in activity specifically toward 5-HT_1A_. In the structural comparison, the indole ring of bufotenine derivatives in 5-HT_1A_ is rotated about 26.6° relative to 5-HT_2A_, altering the local polarity effect (Fig. 4F).

### Structures of therapeutic drugs bound to 5-HT_1A_

**Fig. 5.**
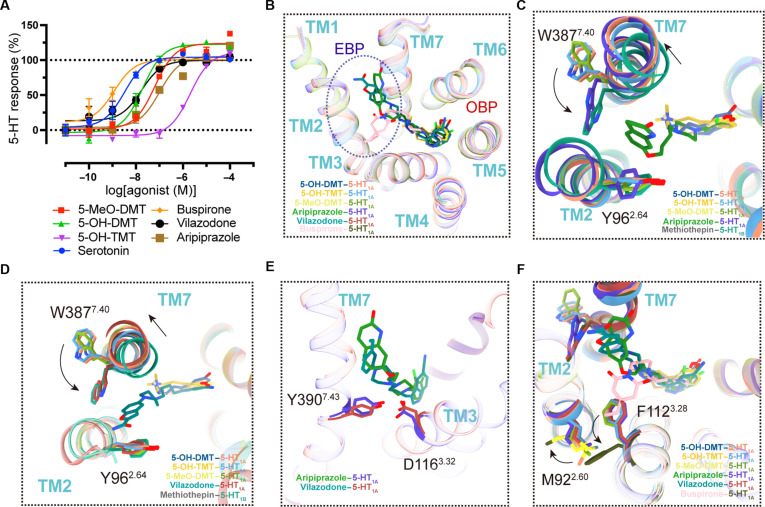
Structural and functional comparison of bufotenine derivatives and clinical 5-HT_1A_ medications at 5-HT_1A_. (A) 5-HT_1A_–Gi GloSensor assays of 5-HT, 5-MeO-DMT, 5-OH-DMT, 5-OH-TMT, vilazodone, aripiprazole, and buspirone. All signaling experiments were performed in triplicate and were averaged from 3 independent experiments. Data were normalized against 5-HT, and errors bars denote the standard error of the mean (SEM). (B) Comparison of the positioning of bufotenine derivatives and therapeutic 5-HT_1A_ medications inside the 5-HT_1A_R pocket. (C) The quinolone segment of aripiprazole influences alterations in the side chain of the N387^7.40^ residue and the displacement of TM7 inside the 5-HT_1A_ extended binding pocket. (D) Vilapatone’s benzofuranocarboxamide group affects the displacement of TM7 inside the 5-HT_1A_ extended binding pocket as well as changes to the side chain of the N387^7.40^ residue. (E) The *N*-aryl substituent of aripiprazole and the *N*-alkyl substituent of vilazodone attach to the orthosteric binding pocket (OBP). (F) The azacyclodecane-7,9-dione component of buspirone curves into the interstice between TM2 and TM3. The black arrows indicate the trajectory of the receptor helix or the residues side chain’s migration. EBP, extended binding pocket.

5-HT_1A_ serves as a therapeutic target for the anxiolytic medication buspirone and the antidepressant vilazodone, both of which are often utilized in clinical practice. In the comparison of commonly used clinical drugs, we conducted functional experiments. The findings indicated that the EC_50_ values of 2 buprenorphine derivatives were approximately comparable to those of clinically utilized drugs (Fig. 5A). All 6 compounds reside in the OBP; however, they exhibit varying depths and an extended binding pocket (PDB: 7E2Z, 8FYL, and 8FYX) (Fig. 5B). Buspirone had the highest potency, with an EC_50_ of 1 nM. 5-MeO-DMT, 5-OH-DMT, aripiprazole, and vilazodone exhibited comparable affinities, with EC_50_ spanning around 10 to 100 nM. Unlike tryptamine derivatives, aripiprazole may interact with the N387^7.40^ residue site on TM7, a unique binding site believed to stabilize the quinolone segment of aripiprazole inside the 5-HT_1A_ extended binding pocket (Fig. 5C). The vilazodone molecule contains an indole core that is connected to the OBP. The W387^7.40^ residue on TM7 and the Y96^2.64^ residue on TM2 exhibit π–π interactions with the benzofuranocarboxamide group of vilapatone (Fig. 5D). Aripiprazole and exrazolone possess similar OBP structures, characterized by the inward rotation of the indole ring at W387^7.40^ and the phenolic hydroxyl group at Y96^2.64^, hence augmenting polar contacts, in stark contrast to the tryptamine derivatives. The benzofurancarboxamide moiety of vilazodone extends toward the extracellular site, whereas the piperazine part is positioned closer to the extracellular area compared to other compounds. The presence of this characteristic may be attributed to the distinct binding configurations of vilazodone and aripiprazole. In particular, the *N*-aryl substituent of aripiprazole and the *N*-alkyl substituent of vilazodone attach to the OBP, causing D116^3.32^ and Y390^7.43^ to interact with the identical piperazine in both drugs (Fig. 5E). The prolonged pocket in the structure of buspirone is maintained by the involvement of M92^2.60^, A93^2.61^, and C187^ECL2^ at the TM2 location. Buspirone has a distorted structural conformation, whereby the azacyclodecane-7,9-dione component curves into the interstice between TM2 and TM3, causing the side chains of M92^2.60^ and F112^3.28^ to rotate outward accordingly (Fig. 5F). Within the context of the OBP, the piperazine component of buspirone engages with D116^3.32^ in a way akin to aripiprazole (Fig. S5D). Specifically, the pyrimidine portion of buspirone is positioned in close proximity to TM3 and TM5, resulting in mostly hydrophobic interactions (Fig. S5E). In general, we see that although they have distinct structures, vilazodone, aripiprazole, and buspirone have comparable orientations in the central binding pocket. The aromatic piperazine substituents of these compounds are mostly stabilized by phenylalanine residues in TM6 (F361^6.51^ and F362^6.52^) (Fig. S5F). Therefore, it may be inferred that bufotenine derivatives have pharmacological actions that are comparable to those of antidepressants, as they display a similar binding pattern in the OBP.

### 5-OH-DMT and 5-MeO-DMT exhibited similar improvement against depressive-like behaviors through 5-HT_1A_R

**Fig. 6.**
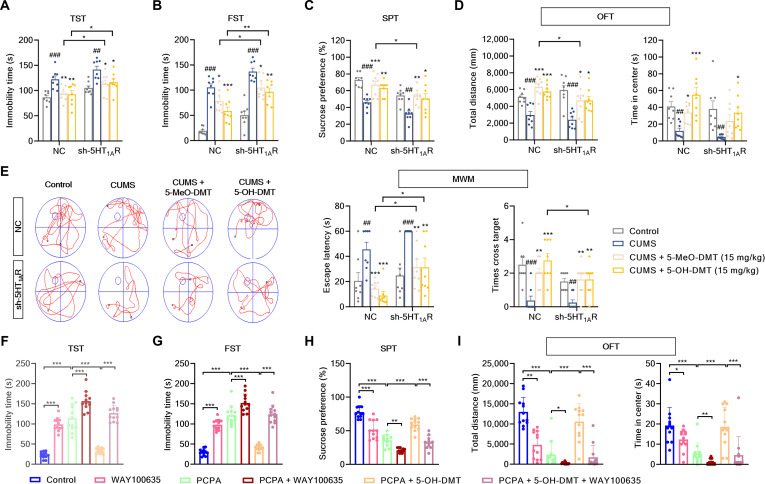
5-OH-DMT and 5-MeO-DMT exhibited similar improvement against depressive-like behaviors through 5-HT_1A_R. The (A) tail suspension test (TST), (B) forced swimming test (FST), (C) sucrose preference test (SPT), (D) open-field test (OFT), and (E) Morris water maze test (MWM) were performed in negative control (NC) and sh-5-HT_1A_R mice treated with or without chronic unpredictable mild stress (CUMS), 5-MeO-DMT, and 5-OH-DMT (*n* = 8). (F) TST, (G) FST, (H) SPT, and (I) OFT were performed in mice treated with or without 4-chloro-dl-phenylalanine (PCPA), 5-OH-DMT, and the 5-HT_1A_R antagonist WAY100635 (*n* = 12). All quantification data are presented as mean ± SEM. ^#^*P* < 0.05 and ^##^*P* < 0.001 versus the control group; *P < 0.05, **P < 0.01, and ***P < 0.001 versus the model group.

We explored the therapeutic effects of 5-MeO-DMT and 5-OH-DMT on depression and the involvement of 5-HT_1A_R by stereotactically injecting AAV with Syn-eGFP-sh-5-HT_1A_R to knock down 5-HT_1A_R in the hippocampus (Figs. S8 and S9). Here, we focused on 5-HT_1A_R in the mouse hippocampus, because the hippocampus is of particular importance in regulating mood and adult neurogenesis, as well as the highest levels of 5-HT_1A_R in the hippocampal area [43–45]. Mice were subjected to chronic unpredictable mild stress (CUMS) to model depression, and their condition was assessed through behavioral tests (Fig. S8A). CUMS prolongs immobility periods in both negative control (NC) and sh-5-HT_1A_R mice during the tail suspension test (TST) and forced swimming test (FST). In NC mice, 5-MeO-DMT and 5-OH-DMT significantly reduce immobility time in both TST (5-MeO-DMT: 80.21%; 5-OH-DMT: 83.06%) and FST (5-MeO-DMT: 31.03%; 5-OH-DMT: 54.46%). However, in sh-5-HT_1A_R mice, the effects are diminished, with 5-MeO-DMT showing 77.37% efficiency in TST and 37.07% in FST and 5-OH-DMT showing 68.76% efficiency in TST and 46.50% in FST (Fig. 6A and B and Table S3). In the sucrose preference test, both 5-MeO-DMT and 5-OH-DMT significantly counteract the reduction in sucrose preference induced by CUMS. However, in comparison to NC mice, only sh-5-HT_1A_R mice treated with 5-MeO-DMT show a decrease in sucrose preference (Fig. 6C and Table S3). The open-field test was conducted to assess anxiety-like behaviors. Results indicated that both 5-MeO-DMT and 5-OH-DMT increased the total distance traveled and time spent in the center, regardless of 5-HT_1A_R knockdown. However, 5-MeO-DMT reduced total distance after 5-HT_1A_R was knocked down (Fig. 6D and Table S3). Spatial learning and memory were assessed using the Morris water maze test. In NC and sh-5-HT_1A_R mice, 5-MeO-DMT and 5-OH-DMT decrease escape latencies (5-MeO-DMT: 124.47% efficiency; 5-OH-DMT: 146.30% efficiency) and increase target crossing times (5-MeO-DMT: 76.47% efficiency; 5-OH-DMT: 111.76% efficiency). sh-5-HT_1A_R mouse treatment with these 2 bufotenine derivatives increases escape latencies (efficiency of 5-MeO-DMT: 81.41%; efficiency of 5-OH-DMT: 81.93%), but only 5-OH-DMT reduces times across target (efficiency: 110.00%), compared to NC mice (Fig. 6E and Table S3). The findings indicate that both 5-MeO-DMT and 5-OH-DMT similarly alleviate CUMS-induced depressive-like and anxiety-like behaviors, as well as enhance learning and memory via 5-HT_1A_R. Furthermore, it is worth noting that in contrast to 5-MeO-DMT, 5-OH-DMT treatment resulted in somewhat reduced head-twitch response (HTR) and incremental horizontal locomotor activity (HLA) in mice (Fig. S10), indicating diminished hallucinogenic potential while preserving therapeutic antidepressant effects.

To further validate that 5-OH-DMT elicits antidepressant-like action via 5-HT-dependent mechanism, we generated a 4-chloro-dl-phenylalanine (PCPA)-induced mouse model of depression and anxiety and gave treatment with or without 5-OH-DMT (15 mg/kg) and 5-HT_1A_R antagonist WAY100635 before behavioral tests (Fig. S8B). As expected, compared with the control or model group, WAY100635 significantly increased immobility time in both TST and FST. In comparison, mice treated with 5-OH-DMT exhibited shorter immobility durations in these tests. However, the addition of WAY100635 to the PCPA and 5-OH-DMT group also prolonged the immobility time in both TST and FST (Fig. 6F and G). WAY100635 reduced sucrose preference compared to the control or model group. Conversely, treatment with 5-OH-DMT increased sucrose preference in mice, while this increase was reversed by WAY100635 (Fig. 6H). In addition, mice treated with WAY100635 showed less total distance traveled and less time spent in center compared to the control or PCPA mice in the open-field test. By contrast, 5-OH-DMT dramatically increased both the total distance and time spent in the center, whereas the administration of WAY100635 in the PCPA and 5-OH-DMT group significantly recovered these changes (Fig. 6I). These results reconfirmed that 5-OH-DMT ameliorates depressive-like behaviors through 5-HT_1A_R.

## Discussion

The role of 5-HT_1A_R signaling in the therapeutic efficacy of antidepressants is well established [46–48]. Here, we provide the molecular structures of bufotenine derivatives in complex with 5-HT_1A_R, alongside a comprehensive analysis of their signaling behaviors at this receptor. This approach illuminates the precise relationship between the chemical configurations of bufotenine derivatives and their functional effects. Notably, bufotenine derivatives exhibit a more inclined orientation within the binding pocket compared to serotonin, with structural differences across binding sites influencing their interaction profiles. The structure–activity relationships reveal distinct contributions of key functional groups to 5-HT_1A_R binding and activation. The hydroxyl group at the 5-position of the indole ring serves as a critical hydrogen bond donor, potentially interacting with polar residues such as D116^3.32^ or S199^5.42^ within the OBP, thereby enhancing binding affinity and stabilizing the ligand–receptor complex [49–51]. This hydroxyl substitution distinguishes bufotenine derivatives from other tryptamines and may account for their unique pharmacological profile. Methoxy substitutions introduce additional steric bulk and electron-donating properties that can modulate receptor selectivity [52–54]. The methoxy group’s oxygen atom may participate in weak hydrogen bonding interactions with backbone amides or hydroxyl-containing residues, while its methyl component influences overall lipophilicity and membrane permeability [55]. Importantly, methoxy groups at different positions (e.g., 4-methoxy vs. 7-methoxy) create distinct binding orientations within the pocket, potentially explaining subtype selectivity between 5-HT_1A_ and other serotonin receptors [37,56]. *N*,*N*-Dimethyl substitutions on the ethylamine side chain significantly impact both binding kinetics and efficacy. The methyl groups increase the basicity of the amine nitrogen, enhancing ionic interactions with the conserved D116^3.32^ that anchors most aminergic ligands. Additionally, the steric hindrance imposed by N-methylation may restrict conformational flexibility, favoring specific receptor conformations associated with particular signaling pathways. This could explain why certain *N*-methyl derivatives show biased signaling toward G-protein activation over β-arrestin recruitment, contributing to their therapeutic potential with reduced side effects [57]. These variations in structure modulate the activity and affinity of bufotenine derivatives at 5-HT_1A_R and 5-HT_2A_R.

We speculate that 5-OH-DMT’s unique hydrogen bonding with I189^ECL2^ reduces β-arrestin recruitment, which may be associated with its low hallucination risk; however, this mechanistic explanation requires further validation through in-depth studies of receptor trafficking dynamics and electrophysiological consequences. Fluorescence recovery after photobleaching studies could quantify 5-HT_2A_R internalization rates and recycling kinetics following 5-OH-DMT treatment compared to classical hallucinogens. Additionally, whole-cell patch-clamp recordings in cortical pyramidal neurons would reveal the temporal profile of receptor activation and downstream effector coupling. Brain slice electrophysiology could assess network-level effects, particularly examining gamma oscillations in layer 2/3 cortical circuits that correlate with hallucinogenic experiences. Such studies would provide mechanistic validation of our molecular findings and establish the functional significance of reduced β-arrestin signaling. Furthermore, 2-photon calcium imaging in awake behaving animals could bridge molecular mechanisms with behavioral phenotypes, ultimately supporting the therapeutic potential of 5-OH-DMT derivatives.

Importantly, bufotenine derivatives display unique conformations and distinct structural attributes relative to traditional antidepressants, enabling the development of structure-based bufotenine derivatives with selective 5-HT_1A_R activity and reduced affinity for 5-HT_2A_Rs. Given that hallucinogenic effects are predominantly associated with 5-HT_2A_R activation, the comparatively moderate hallucinogenic potential of 5-HT_1A_R is notable. Since 5-MeO-DMT clearly targets 5-HT_2A_R, we focused on 5-OH-DMT in our in vivo experiments. Bufotenine derivatives demonstrate a significant enhancement in antidepressant efficacy in the CUMS model or PCPA model. Knockdown of hippocampal 5-HT_1A_R partly abolishes the effect of 5-OH-DMT in improving CUMS-induced depressive-like behaviors. However, the 5-HT_1A_R antagonist WAY100635 almost completely abolishes the antidepressant effect of 5-OH-DMT in the PCPA model. These differential effects might be explained by that except for the hippocampus, other brain regions also participate in 5-OH-DMT-exerted antidepressant effect. Moreover, PCPA-induced depression is clearly due to serotonin depletion, while the mechanism of CUMS-induced depression is more complex. Our findings confirm bufotenine derivatives as full agonists at 5-HT_1A_R. These insights underscore the potential of bufotenine derivatives as a promising avenue for targeted antidepressant development. Further study is needed to test 5-HT_1A_ agonists such as buspirone or vilazodone in biological experiments to support any competitive advantage for bufotenine derivatives.

Psychedelics are increasingly recognized as potential treatments for neuropsychiatric disorders. Classical psychedelics primarily target 5-HT_2A_R on cortical pyramidal neurons, influencing their hallucinogenic and subjective effects [58]. However, whether 5-HT_2A_R activation is indispensable for the neuroplastic and therapeutic potential of psychedelics remains debated. Growing evidence suggests that psychedelics exhibit complex polypharmacological profiles that extend beyond their 5-HT_2A_R-mediated hallucinatory actions. Receptors like TrkB, 5-HT_2C_, and 5-HT_1A_ are increasingly acknowledged as crucial binding sites that enhance the plasticity and therapeutic effectiveness of psychedelics, separate from their hallucinogenic effects [59]. 5-HT_1A_R is implicated in the behavioral effects of tryptamine hallucinogens, especially 5-MeO-DMT, aligning with some findings from our study [37]. Psychedelics are thus likely to act through multiple pathways, with the specific receptor profiles engaged by each compound potentially optimizing their therapeutic utility across various disorders. Our findings demonstrate that bufotenine derivatives exert antidepressant effects primarily through 5-HT_1A_R.

A diverse array of chemically distinct compounds is essential for developing new drugs targeting 5-HT_1A_R, with tailored efficacy, activity, and selectivity profiles. Each psychedelic compound can elicit unique introspective effects, shaped by genetic differences that influence drug metabolism and individual responses. Although psychedelics often act on multiple molecular targets, their specific effects vary with chemical structure. Thus, identifying the optimal psychedelic-inspired treatment for a given condition is not always straightforward. Expanding the chemical landscape by considering both the subjective effects and structural diversity of these compounds is crucial for advancing personalized therapeutics.

## Conclusion

In conclusion, our integrated structural and functional analyses reveal how bufotenine derivatives—5-MeO-DMT, 5-OH-DMT, and 5-OH-TMT—selectively engage and activate 5-HT_1A_R at high resolution. These compounds demonstrate robust antidepressant efficacy in vivo while exhibiting attenuated hallucinogenic properties, largely attributed to their specific modulation of 5-HT_1A_-mediated G-protein signaling. Collectively, our findings establish a mechanistic framework for the rational design of 5-HT_1A_-targeted therapeutics and support the potential of bufotenine derivatives as promising candidates for the development of safer and more effective antidepressant and anxiolytic drugs.

## Methods

### Creating compounds through chemical reactions

Compound 1 (5-MeO-DMT) was synthesized by reacting (4-methoxyphenyl)hydrazine hydrochloride (5.0 mmol) with 4,4-diethoxy-*N*,*N*-dimethyl-1-butylamine (5.25 mmol) in water and sulfuric acid under N_2_ at 37 °C for 14 h. The mixture was basified (pH 10) and extracted with ethyl acetate. The crude product was purified via silica gel chromatography (dichloromethane [DCM]:methanol [MeOH] = 30:1) to yield a brown-yellow paste (684.4 mg, 62.7%). ^1^H nuclear magnetic resonance (NMR) (500 MHz, CDCl_3_) *δ* 7.94 (s, 1H), 7.24 (d, *J* = 8.8 Hz, 1H), 7.05 (d, *J* = 2.4 Hz, 1H), 7.00 (d, *J* = 2.4 Hz, 1H), 6.85 (dd, *J* = 8.8, 2.4 Hz, 1H), 3.86 (s, 3H), 2.93 to 2.88 (m, 2H), 2.65 to 2.61 (m, 2H), 2.34 (s, 6H). ^13^C NMR (500 MHz, CDCl_3_) *δ* 154.07, 131,56, 127.74, 122.58, 112.00, 100.67, 59.79, 56.00, 44.97, 23.21.

Compound 2 (5-OH-DMT) was prepared using 5-benzyloxyindole (10 mmol) and oxalyl chloride (20 mmol) in anhydrous diethyl ether at room temperature for 30 min. The red solid was collected by filtration, washed with ether, and dried under vacuum to afford 5-benzyloxy-3-indol-glyoxylylchloride. An aqueous NaOH solution was added dropwise to a suspension of 5-benzyloxy-3-indol-glyoxylylchloride (10 mmol) and dimethylamine hydrochloride (42.9 mmol) in ether at 25 °C for over 30 min. The crude product was obtained by filtration. The product was hydrogenated (10% Pd/C, H_2_, 24 h), filtered, and purified via silica gel (DCM:MeOH = 30:1) to give 6.1 g of the product (92% yield). The product (35 mg, 0.15 mmol) was reduced with LiAlH_4_ (9 mmol) in tetrahydrofuran at 0 °C, refluxed for 4 h, and then stirred at 40 °C for 10 h. After NaOH quenching and workup, column chromatography (CH_2_Cl_2_:MeOH = 10:1) gave compound 2 (5-OH-DMT) as a yellow-brown oil (85% yield). ^1^H NMR (dimethyl sulfoxide-*d*_6_ [DMSO-*d*_6_], 500 MHz) and ^13^C NMR confirmed the structure [60].

Compound 3 (5-OH-TMT) was obtained by methylating compound 2 (20 mmol) with methyl iodide (120 mmol) in MeOH under nitrogen for 24 h, followed by silver chloride treatment to remove iodide. After extraction and chromatography (DCM:MeOH = 20:1), 3.4 g of the product was isolated (47.9% yield). ^1^H NMR (DMSO-*d*_6_, 500 MHz) and ^13^C NMR supported successful quaternization [60].

### Construction

To achieve insert cell expression, Synbio Technologies optimized the human wild-type 5-HT_1A_ gene codons. A thermally stable apocytochrome b562 RIL was inserted at the N-terminal of human 5-HT_1A_R to boost receptor expression [61]. The proteins included affinity markers like the N-terminal Flag and His tags. It was shown that L125W alterations in 5-HT_1A_ improved thermal stability [62]. The Vazyme Biotech ClonExpress II One-Step Cloning Kit cloned 5-HT_1A_R into pFastBac. The receptor’s N terminus was 24 residues short. Dominant-negative human Gαi1 was produced with mutations S47N, G203A, A326S, and E245A using site-directed mutagenesis. Mutations increase the dominant-negative impact by destroying the βγ subunits’ stabilizing salt bridge [40]. Cloned pFastBac vectors include human dominant-negative Gαi1 (DNGαi1), Gβ1, and Gγ2 genes. A modified pFastBac vector cloned scFv16. The vector’s N-terminus includes the GP67 secretion signal peptide. While being built, insect cell expression vectors created these structures.

### Cell culture and protein expression

At 27 °C, High Five insect cells (Hi5, Invitrogen) were grown in ESF 921 serum-free medium (Expression Systems). Agilent HEK 293T cells were cultured in Thermo Fisher’s Dulbecco’s modified Eagle medium (DMEM) with 10% fetal bovine serum (FBS). A room with regulated humidity, 5% CO_2_, and 37 °C temperature housed the cultivation. The Bac-to-Bac Baculovirus Expression System from Thermo Fisher developed baculoviruses. Human genes 5-HT_1A_, DNGαi1, Gβ1, Gγ2, and scFv16 were coexpressed in High Five cells using Expression Systems’ baculovirus technique. Cell cultures were grown to 2 to 3 million cells/ml and evenly treated to 5 baculoviruses. After 48 h of infection, centrifuged cells were kept at −80 °C for examination.

### Purification of protein complexes

Cryopreserved cells were lysed in buffer (20 mM Hepes pH 7.5, 100 mM NaCl, 10% glycerol, 10 mM MgCl_2_, and 5 mM CaCl_2_) with EDTA-free protease inhibitors. After Dounce homogenization, 5-HT_1A_–Gi–scFv16 complexes were formed by adding 25 mU/ml apyrase and 100 μM ligand at room temperature for 1.5 h. Membranes were solubilized in 0.5% lauryl maltose neopentyl glycol and 0.1% cholesteryl hemisuccinate at 4 °C for 2 h and clarified by ultracentrifugation (30,000 rpm, 30 min). The supernatant was incubated with Talon beads for 2 h at 4 °C. After washing with low imidazole buffer, bound complexes were eluted with 250 mM imidazole. The eluate was concentrated using a 100-kDa cutoff filter and purified by size-exclusion chromatography (Superose 200 Increase 10/300 GL). Eluted fractions were analyzed by sodium dodecyl sulfate–polyacrylamide gel electrophoresis (Figs. S1 to S3). For cryo-EM investigation, receptor–Gi-protein complex samples were combined and condensed.

### Cryo-EM data acquisition

Purified 5-MeO-DMT-, 5-OH-DMT-, and 5-OH-TMT-bound 5-HT_1A_–Gi–scFv16 complexes (12, 10, and 13 mg/ml) were vitrified on glow-discharged Quantifoil grids using a Vitrobot at 4 °C. Cryo-EM data were acquired on a Titan Krios G4 (300 kV) with a Falcon 4i detector and Selectris X, collecting 13,026, 5,233, and 5,387 electron event representation (EER) movies at 0.73 Å/pixel, 10 e^−^/Å^2^/s, with defocus from −0.8 to −1.8 μm.

### Image processing and 3-dimensional reconstruction

Motion correction was performed using MotionCor2 [63,64], and contrast transfer function estimation was done with CTFFIND [65] in cryoSPARC. Low-quality micrographs (>4.0 Å or carbon contaminated) were excluded. Particle picking (blob, Topaz, template) and 2-dimensional classification identified high-quality particles for ab initio reconstruction and heterogeneous refinement. After merging and de-duplication, 180,694, 195,003, and 139,905 particles were refined for the 5-MeO-DMT, 5-OH-DMT, and 5-OH-TMT complexes, respectively, yielding global resolutions of 2.63, 2.54, and 2.59 Å, respectively. Local refinement on the receptor achieved 2.75, 2.55, and 2.74 Å, respectively, followed by DeepEMhancer [66] post-processing (Figs. S1 to S3).

### Model building and refinement

The density maps were fitted with structural models of 5-HT_1A_, Gαi, Gβ, Gγ, and scFv16 from PDB entries 7E2Z and 7YS6 (Fig. S4) [41]. Initial fitting into locally refined receptor maps was performed in UCSF Chimera [67], followed by iterative manual adjustments in COOT [68] and automated refinement in PHENIX [69]. G protein and receptor models were further fitted into B-factor-sharpened maps, refined in COOT and PHENIX, and subjected to additional reconstruction using ISOLDE [70] and PHENIX with torsion restraints. Final models were validated via PHENIX cryo-EM validation (Table S1). Figures were prepared using Chimera, ChimeraX [71], and PyMOL (Schrödinger, LLC.).

### Molecular docking

The molecular docking models were constructed using the 5-HT_2A_ structures (PDB: 7RAN). The models were generated by removing all ligands and water molecules from these structures. The PubChem database (https://pubchem.ncbi.nlm.nih.gov/) was used to download the structures of the small-molecule ligands of interest. At a physiological pH of 7.4 and using AMBER ff 14SB parameters [72], protonation states were assigned using H++ 3.2v (http://newbiophysics.cs.vt.edu/H++/). Then, the Chiron tool was used to decrease energy in the protein structure [73]. At the center of *X*: 130.464, *Y*: 116.935, and *Z*: 87.163, the grid box covers virtually all of the pocket residues; the 5-HT_1A_ ligand docking grid line was *X*: 16.833, *Y*: 12.877, and *Z*: 14.753 (Å). Using AutoDock Vina (version 1.1.2) [74], docking computations were carried out. The ChimeraX software was used to view the docked protein–ligand structures.

### Animals

Adult C57BL/6 male mice (6 to 8 weeks) were purchased from QingLongShan (Nanjing, China). The mice were individually housed at 18 to 22 °C with water and food ad libitum and at a 12:12 h light cycle.

### Ethics statement

All experiments involving animals were conducted according to ethical policies and procedures approved by the Animal Care and Use Committee of Nanjing University of Chinese Medicine (Nanjing, China) (Approval No. A220404).

### Stereotactic injections

Adeno-associated viruses (AAVs) encoding AAV-Syn-eGFP-scramble shRNA (1.26 × 10^13^ viral genomes/ml) and AAV-Syn-eGFP-sh-5-HT_1A_R (1.10 × 10^13^ viral genomes/ml) were generated by OBIO Technology (Shanghai, China). Mice received stereotactic injections into the hippocampal region at the following coordinates relative to the bregma: anterior/posterior, −2 mm; mediolateral, ±1.5 mm; and dorsoventral, −1.8 mm [75]. Animals were injected with either 500 nl of viruses into both hemispheres to breed hippocampus-specific 5-HT_1A_R knockdown (sh-5-HT_1A_R) mice and NC mice. After 3 weeks, the efficiency of postinfection was verified by enhanced green fluorescent protein (eGFP) fluorescence and relative 5-HT_1A_R messenger RNA level. For messenger RNA detection, hippocampal RNA was isolated and first-strand complementary DNA was synthesized using HiScript II Q RT SuperMix. Quantitative real-time polymerase chain reaction (PCR) was performed with ChamQ SYBR qPCR Master Mix in CFX Connect Real-Time PCR Detection System (Bio-Rad, Redmond, USA). The primers used in this study were as follows: 5-HT_1A_R F, CATCGCGCTAGACAGGTACTG; 5-HT_1A_R R, CAATGAGCCAAGTGAGCGAGA; β-actin F, GTGACGTTGACATCCGTAAAGA; and β-actin R, GCCGGACTCATCGTACTCC.

### CUMS model and treatment

Mice were divided into the control or CUMS model group. For the CUMS model group, mice were exposed to randomized stressors at least once a day over 5 weeks, including 24-h water/food deprivation, reversed light/dark cycle, 24-h soiled/empty cage, 24-h cage tilt, 5-h confinement, 5-min cold swimming, and 6-h noise (80 dB). The control group was housed under normal conditions and switched to individual housing only during the reversed light/dark cycle and noise phase. At week 6, CUMS model mice were randomized into subgroups to receive saline solution (vehicle), 15 mg/kg 5-MeO-DMT, and 15 mg/kg 5-OH-DMT for the next 1 week, respectively. The doses chosen were based on previous preclinical experiments by us and others [76,77]. Drugs were dissolved in saline solution containing 0.1% DMSO and disrupted by ultrasound and then immediately administered via intraperitoneal injection. Then, mice with 1-week administration were tested for all behaviors.

### PCPA model and treatment

Mice were randomly divided into the following 6 groups: control, WAY100635, PCPA, PCPA + WAY100635, PCPA + 5-OH-DMT, and PCPA + 5-OH-DMT + WAY100635. Mice in the control group were fed with saline solution containing 0.1% DMSO (vehicle) for 3 d. Mice that were injected subcutaneously with 150 mg/kg PCPA twice daily for 3 d developed symptoms of depression. For pharmacological treatment, mice were treated with 15 mg/kg 5-OH-DMT via intraperitoneal injection for 3 d during PCPA procedures. For WAY100635 treatment, mice were injected intraperitoneally with 1 mg/kg WAY100635 for 3 d. PCPA, 5-OH-DMT, and WAY100635 were dissolved in saline solution containing 0.1% DMSO and disrupted by ultrasound. Then, mice were tested for all behaviors.

### Behavior tests

#### Tail suspension test

Mice were suspended by placing adhesive tape 1 cm from the tail tip. The immobility time was recorded for the last 4 min of the total 6 min.

#### Forced swimming test

Mice were placed in a cylinder filled with water (23 ± 1 °C) for 6 min. The immobility time was recorded during the last 4 min of the test.

#### Sucrose preference test

Mice were habituated with 1% sucrose and water from 2 different bottles for 3 d. These 2 bottles were placed on randomly assigned sides of the cage every day to eliminate side preference. Then, each mouse had free access to 1% sucrose and water from 2 bottles for 3 h. The total volume of liquid consumed from each bottle was measured. Sucrose preference was calculated by the following formula: sucrose preference (%) = sucrose consumption/(sucrose + water consumption) × 100%.

#### Morris water maze test

The water maze was divided into 4 quadrants, and mice were trained to escape from water by swimming to the platform for 4 trials per day with different start points for 4 consecutive days. On day 5, the test was conducted and mice searched freely for 1 min starting from the opposite quadrant of the platform. The latencies to reach the platform and times cross target was recorded using the software Morris.

#### Open-field test

Mice were placed in the center of an open-field box. A total of 5-min video was recorded to observe the locomotor activity of mice. The total distance traveled in the field and the time spent in the center zone by mice were measured.

### Side effect detection

Mice were given 25 mg/kg 5-MeO-DMT or 25 mg/kg 5-OH-DMT by intraperitoneal injection. Then, HTR and HLA were immediately tested. Mice were placed into the observation arena (25 × 25 × 40 cm) under low-light conditions. The number of head-twitch events and total distance were respectively recorded for 10 and 30 min. For the HTR test, the number of head twitches was examined by 2 observers blinded to the treatments via video recordings. For the HLA test, the total distance traveled was measured by using tracking software to process video recordings.

### GloSensor cAMP assay

The downstream Gαi signaling of 5-HT_1A_ was assessed using the GloSensor cAMP assay (Promega). HEK 293T cells were cultured in DMEM with 10% FBS and 1% penicillin–streptomycin at 37 °C and 5% CO_2_. Cells were transfected with 5-HT_1A_ constructs and the GloSensor-22F biosensor at a 3:2 ratio. After 24 h, cells were resuspended in CO_2_-independent medium with 2% GloSensor reagent and plated at 3.5 × 10^5^ cells/ml (20 μl/well) into 384-well plates. After 1-h incubation, forskolin (1 μM) and test ligands were added, and luminescence was immediately recorded using an EnVision plate reader (PerkinElmer).

### Inositol phosphate accumulation assay

The downstream Gαq signaling of 5-HT_2A_ was evaluated using the IP-One HTRF assay (Cisbio). HEK 293T cells were cultured in DMEM with 10% FBS and 1% penicillin–streptomycin at 37 °C, 5% CO_2_. After 24 h of transfection, cells were resuspended in stimulation buffer (8 × 10^5^ cells/ml) and plated at 7 μl/well into 384-well plates. Ligands diluted in buffer (7 μl) were added and incubated for 1 h at 37 °C. IP-One-d2 and anti-IP-One cryptate (3 μl each) were then added, followed by 30-min incubation at room temperature. HTRF signals were measured using an EnVision reader (PerkinElmer).

### Statistical analysis

For all behavioral studies, mice were randomly assigned to the groups. In addition, investigators were blinded to the group until data had been collected. GraphPad Prism 9 was used to analyze all of the functional research data, and the results are provided as mean ± standard error of the mean from at least 3 separate trials. An evaluation of the concentration–response curves was carried out using a logistic equation with 3 parameters. Calculations of pEC_50_ values were performed with the use of the sigmoid 3-parameter equation. In order to assess the significance, a 1-way or 2-way analysis of variance was conducted, followed by Bonferroni’s or Dunnett’s post hoc test. Differences were considered significant when *P* < 0.05 and are shown as *P < 0.05, **P < 0.01, or ***P < 0.001.

## Ethical Approval

All institutional and national guidelines regarding the care and use of animals were adhered to.

## Data Availability

The data supporting this study are available from the corresponding authors upon reasonable request. Cryo-EM density maps have been deposited in the Electron Microscopy Data Bank under accession codes EMD-62593 (5-MeO-DMT), EMD-62595 (5-OH-DMT), and EMD-62594 (5-OH-TMT). The corresponding atomic coordinates are available in the Protein Data Bank under accession codes 9KVG, 9KVI, and 9KVH, respectively. Source data are provided with this paper.
