# Supplementary material for: Structural Pharmacology of Bufotenine Derivatives in Activating the 5-HT1A Receptor for Therapeutic Potential in Depression and Anxiety
Source: Research (Wash D C). 2025 Dec 23;8:0987. doi: 10.34133/research.0987 (PMC12722636; doi:10.34133/research.0987)
Supplement: Supplementary 1 — Figs. S1 to S10 Tables S1 to S3 [file research.0987.f1.zip › 20251019 Supplementary Information.docx]

**Supplementary Information**

**Structural pharmacology of** **bufotenine derivative in activating the 5‑HT_1A_ receptor for therapeutic potential in depression and anxiety**

Shu-jie Li^1,2^^,3,5^, Qing-ning Yuan^1,3,5^, Wen-yuan Wu^1,5^, Zhi-han Chen^1^, Duo Chen^1^, Hong Shan3, Qin-yu Chu4, Wen Hu3, Kai Wu3, Tao Liu3, Yu-yu Zhu1, Li Hou^3^, Jing zhou^1*^, Jia Duan^3*^, Jin-ao Duan^1*^, H. Eric Xu^3*^, Hong-yue Ma^1*^

^1^Jiangsu Collaborative Innovation Center of Chinese Medicinal Resources Industrialization, and Jiangsu Key Laboratory for High Technology Research of TCM Formulae, College of Pharmacy, Nanjing University of Chinese Medicine, Nanjing, 210023, China

2 Department of Traditional Chinese Medicine, Fujian Medical University Union Hospital, Fuzhou 350000, Fujian, China

^3^ State Key Laboratory of Drug Research, Shanghai Institute of Materia Medica, Chinese Academy of Sciences, Shanghai 201203, China

^4^School of Pharmaceutical Science and Technology, Hangzhou Institute for Advanced Study, UCAS, Hangzhou 310024, China

^5^These authors contributed equally

*Correspondence:

Hongyue Ma (hongyuema@njucm.edu.cn), H. Eric Xu (Eric.Xu@simm.ac.cn), Jinao Duan (dja@njucm.edu.cn), Jia Duan (duanjia@simm.ac.cn), Jing Zhou (zhoujing@njucm.edu.cn),


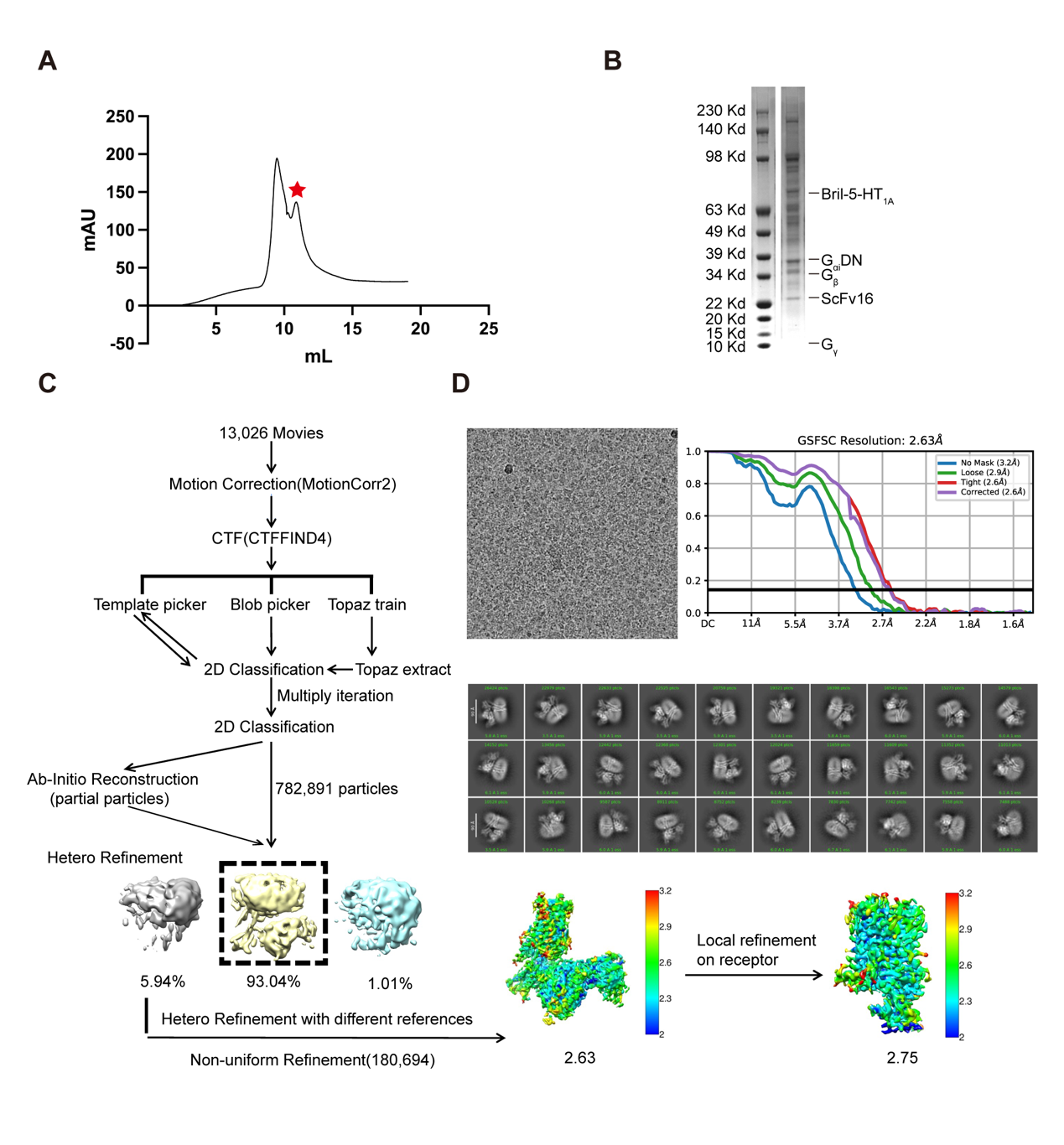


**Fig. S1. 5-MeO-DMT-5-HT_1A_-G_i_- scFv16 complex purification and cryo-EM data processing.** **A-B.** Representative size-exclusion chromatography elution profile and SDS-PAGE analysis. **C.** The flowchart for 5-MeO-DMT-5-HT_1A_-Gi data processing. Details can be found in Methods. **D.** Representative micrographs of 5-MeO-DMT-5-HT_1A_-Gi from CTF estimation (Scale bar, 30 nm).


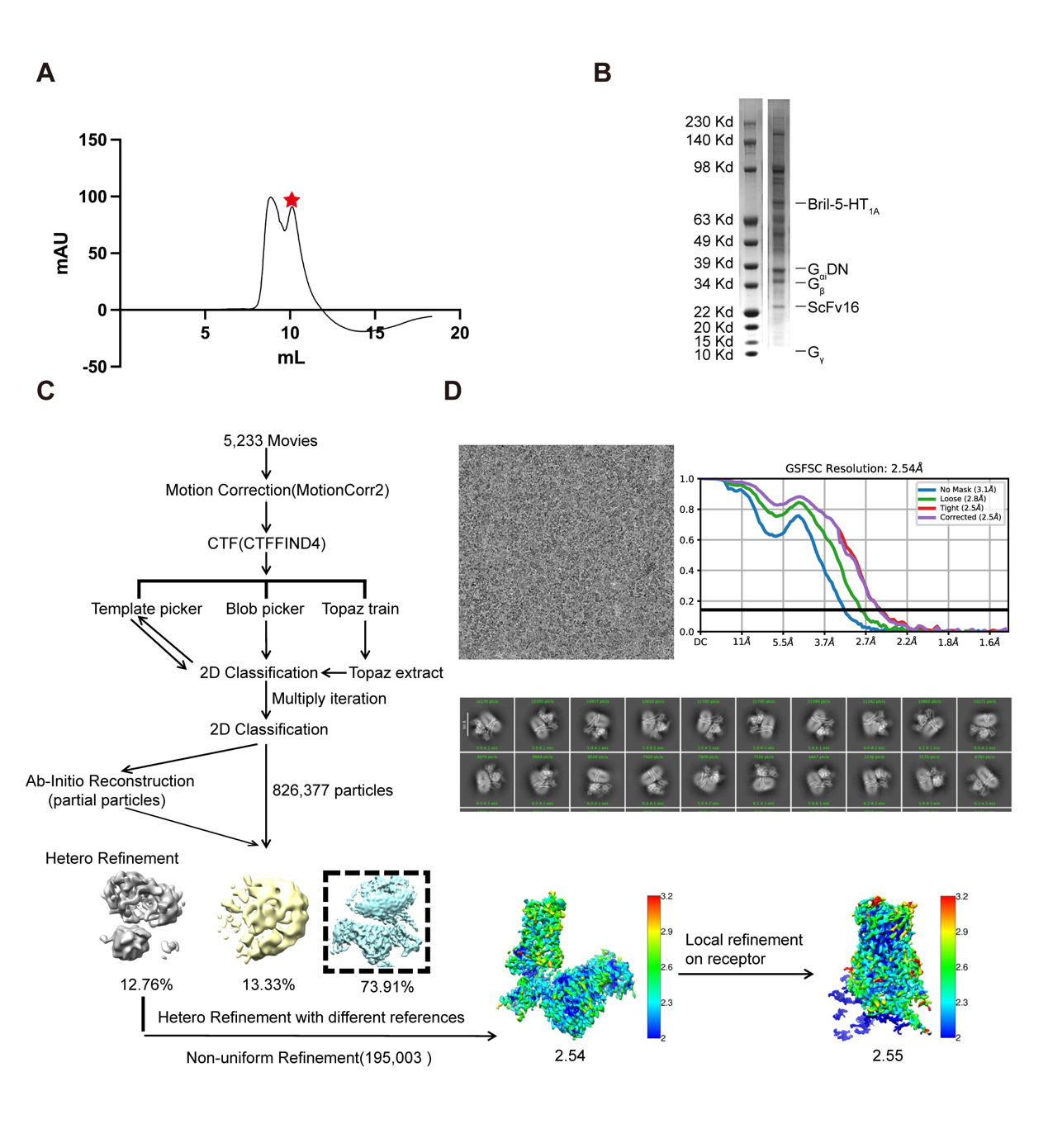


**Fig. S2. 5-OH-DMT-5-HT_1A_-G_i_- scFv16 complex purification and cryo-EM data processing.** **A-B.** Representative size-exclusion chromatography elution profile and SDS-PAGE analysis. **C.** The flowchart for 5-OH-DMT-5-HT_1A_-Gi data processing. Details can be found in Methods. **D.** Representative micrographs of 5-OH-DMT-5-HT_1A_-Gi from CTF estimation (Scale bar, 30 nm).


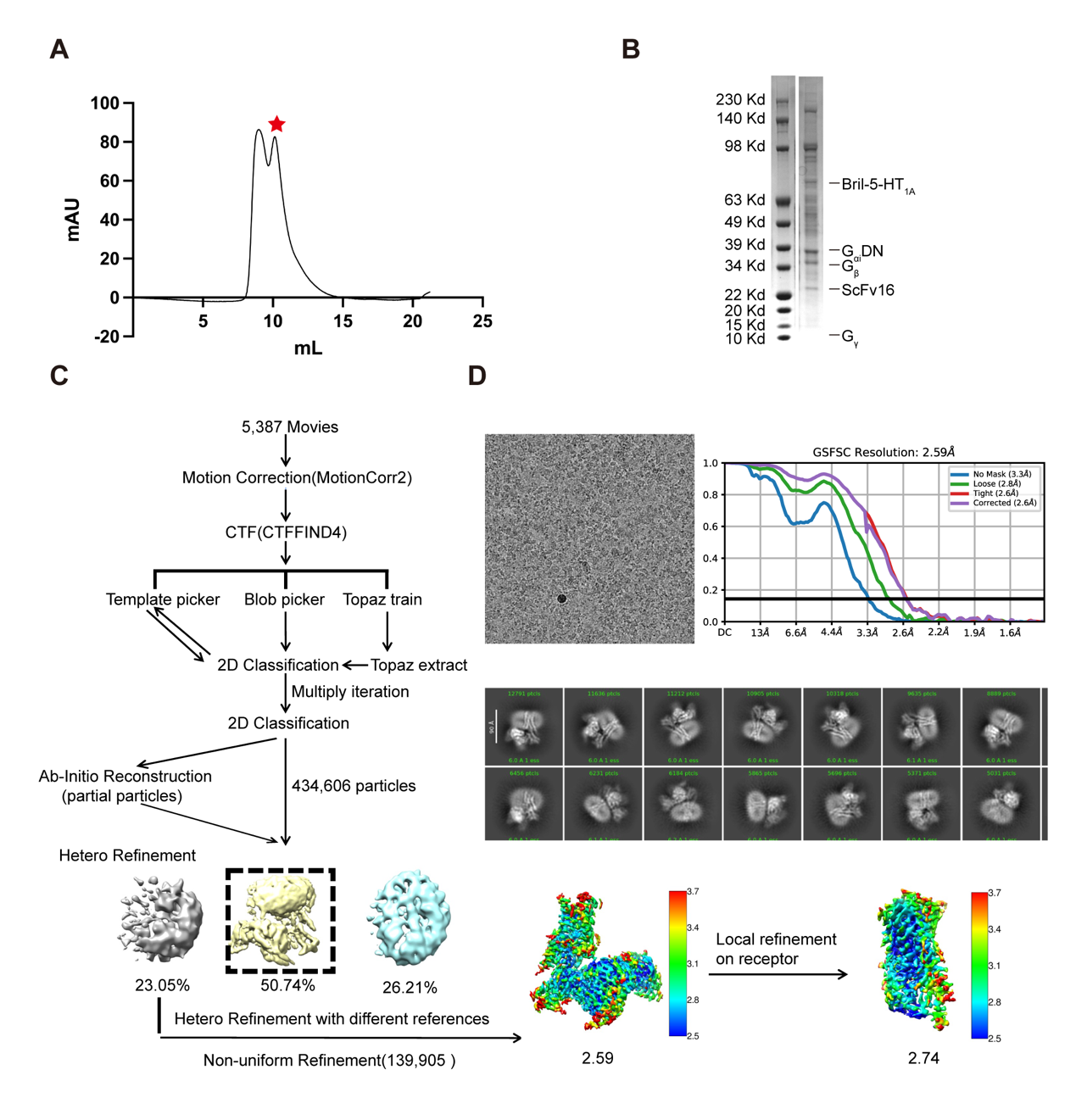


**Fig. S3.** **5-OH-TMT-5-HT_1A_-G_i_-scFv16 complex purification and cryo-EM data processing.** **A-B.** Representative size-exclusion chromatography elution profile and SDS-PAGE analysis. **C.** The flowchart for 5-OH-TMT-5-HT_1A_-Gi data processing. Details can be found in Methods. **D.** Representative micrographs of 5-OH-TMT-5-HT_1A_-Gi from CTF estimation (Scale bar, 30 nm).

**
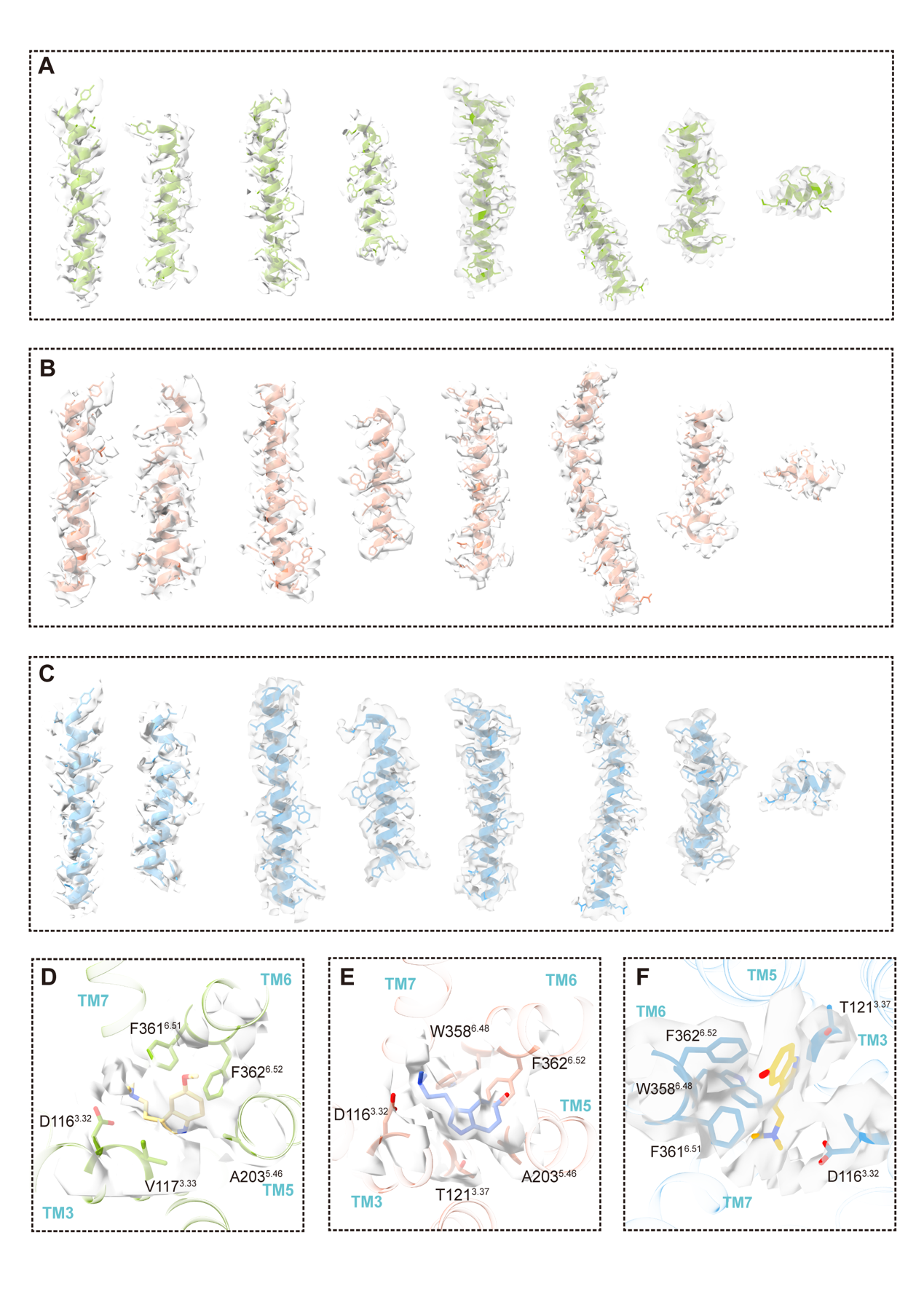
**

**Fig. S4 Representative EM density and coordinate of the 5-**MeO**-DMT-5-HT1A-G_i_ -scFv16, 5-OH-DMT-5-HT_1A_-G_i_- scFv16 and 5-OH-TMT-5-HT_1A_-G_i_-scFv16 complexes.** EM density and model of 5-MeO-DMT-5-HT1A-G_i_ -scFv16 (**A**), 5-OH-DMT-5-HT_1A_-G_i_- scFv16 (**B**), 5-OH-TMT-5-HT_1A_-G_i_-scFv16 (**C**), and key 5-HT_1A_ residues in the binding site of the 5-MeO-DMT (**D**), 5-OH-DMT (**E**), and 5-OH-TMT (**F**).

**
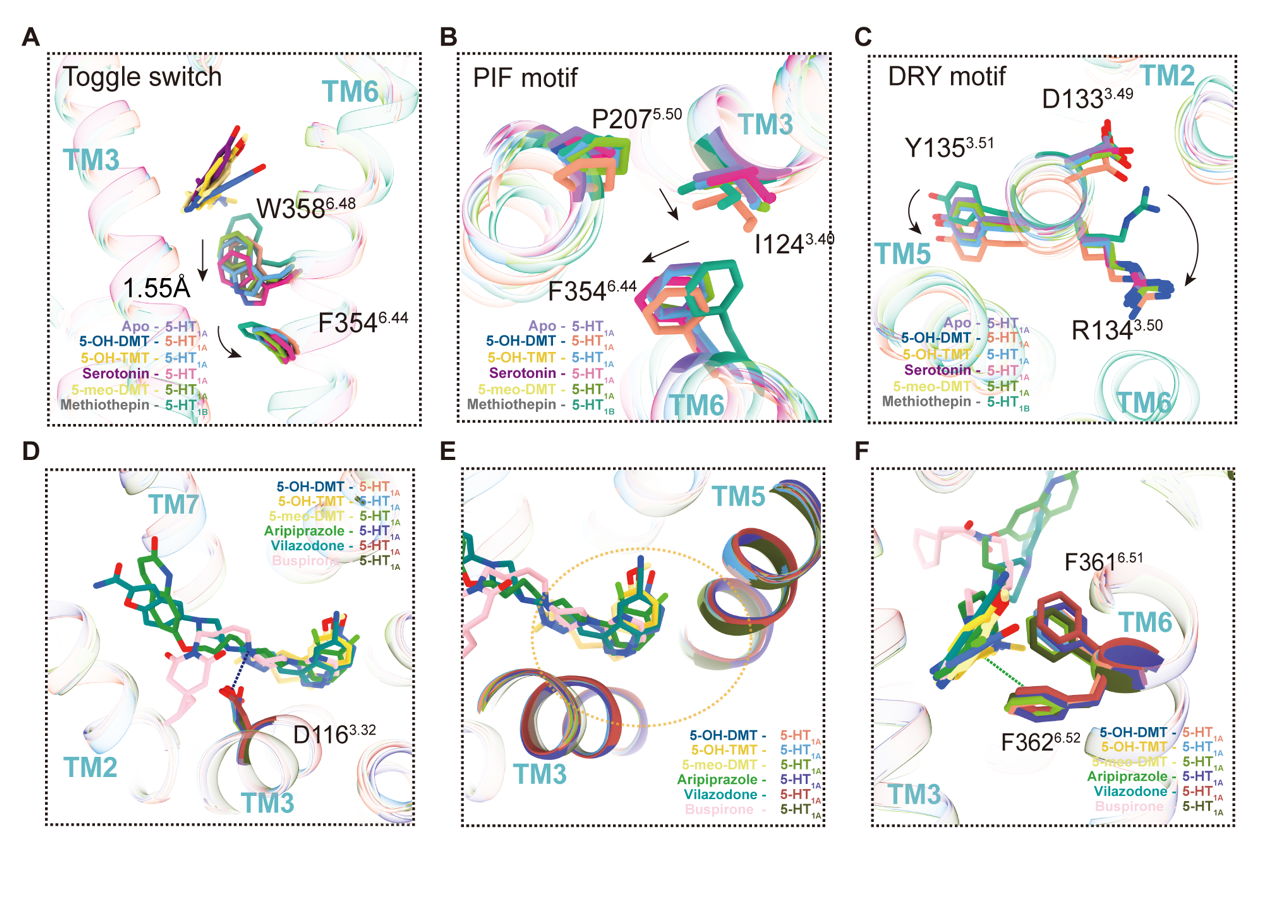
**

**Fig. S5** **Structural features of the active 5-HT_1A_ receptor engaged by bufotenine derivatives and their Structural comparison with clinically used 5-HT_1A_ medications.** A-C. Conformational changes of the conserved “micro-switches” upon receptor activation. Toggle switch (**A**), PIF motif (**B**), and DRY motif (**C**). The outward movement of TM7 of the active receptor and the conformational changes of residue side chains are highlighted as black arrows. **D.** Buspirone engages with D1163.32 like aripiprazole. **E.** The pyrimidine portion of buspirone is positioned in close proximity to TM3 and TM5. **F.** The aromatic piperazine substituents of these compounds are mostly stabilized by phenylalanine residues in TM6. (Dotted blue line: Hydrogen Bonds; Dotted green line: π-π Interactions.)


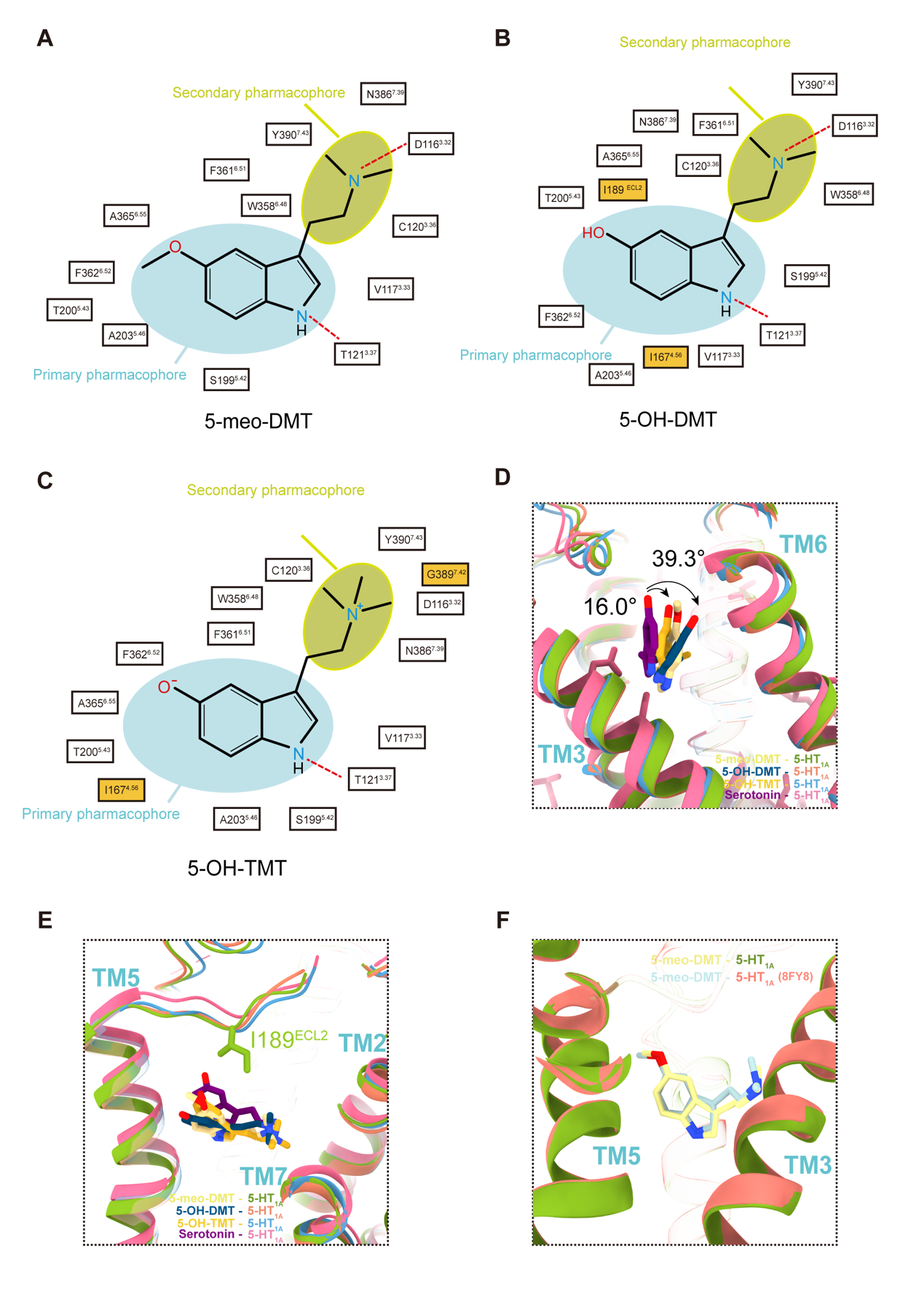


**Fig. S6 Comparative analysis of bufotenine derivatives and ligand interactions within the OBP of 5-HT_1A_. A-E.** Structural comparison of drug-binding poses of 5-MeO-DMT, 5-OH-DMT, and 5-OH-TMT determined in this study. The bufotenine derivatives relies on two key chemical elements: the indole ring and the ethylamine side chain. All compounds assume similar overall poses in the OBP of 5-HT_1A_. **F.** Structural comparison of the 5-MeO-DMT–5-HT_1A_ complex with the cryo-EM structure (PDB: 8FY8).


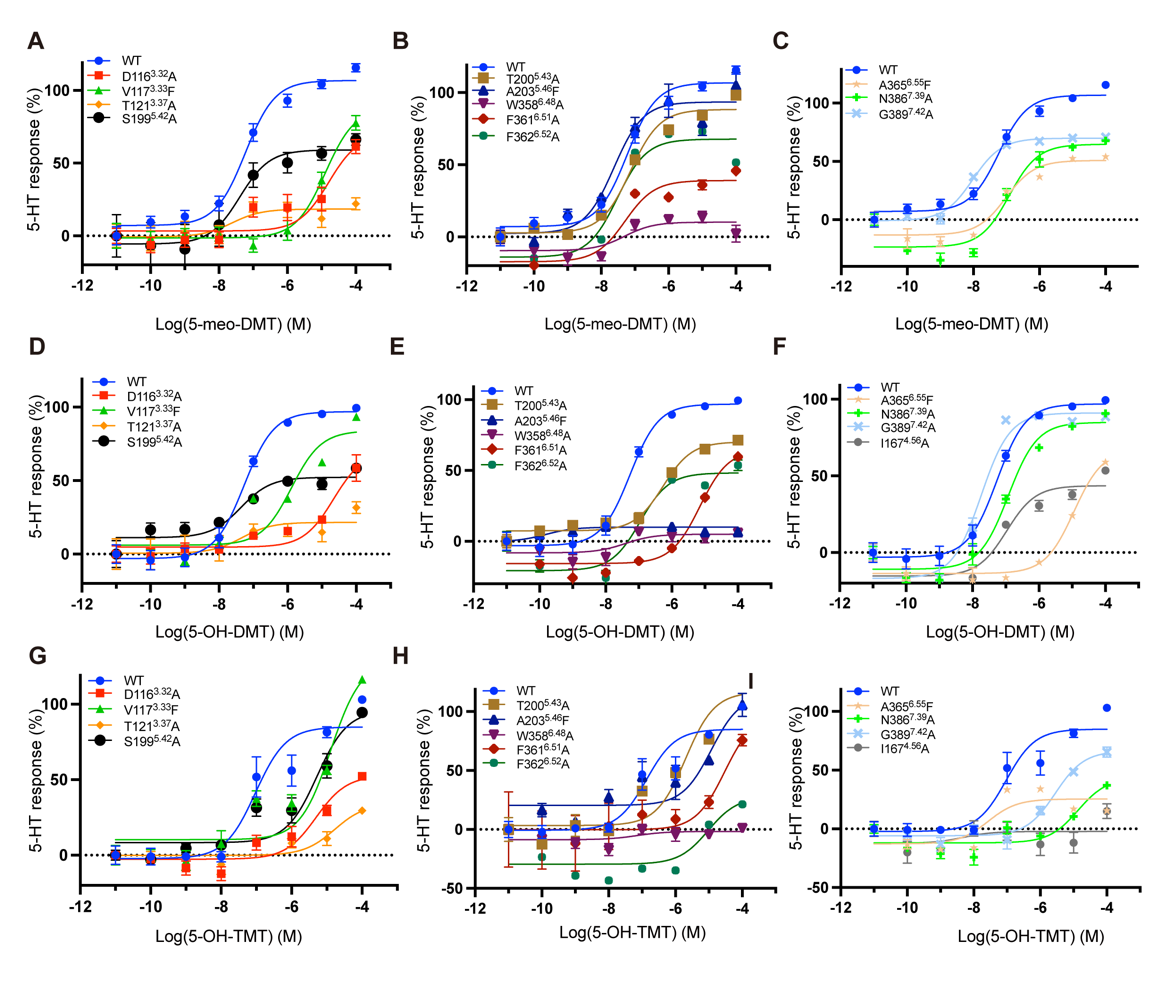


**Fig. S7 5-MeO-DMT, 5-OH-DMT and 5-OH-TMT response curves on WT and mutant** **5-HT_1A_.** WT or mutant were transfected into HEK293T cells and Glosenser cAMP assay were measured to reflect the activity of 5-HT_1A_R. The response data was normalized by WT receptor within each individual experiment, with the basal activity for WT as 0, while the fitted *Emax* of WT as 100. Three independent experiments were conducted (n=3). The representative concentration-response curves were shown. Source data are provided as a Source Data file.

**
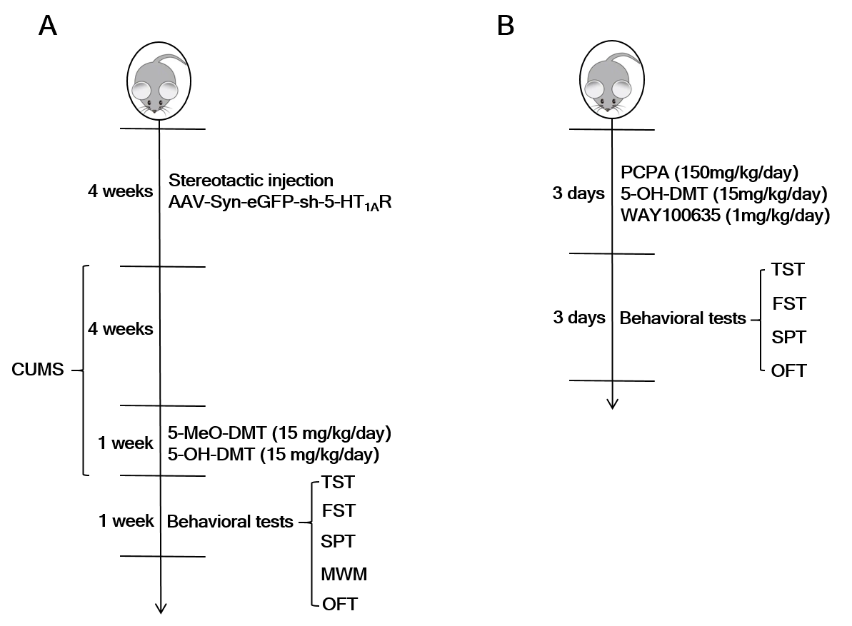
**

**Fig. S8 Schematic diagrams of animal experiments. A**. Schematic diagram of NC and sh-5-HT_1A_R mice with CUMS depression model and treatment. **B.** Schematic diagram of PCPA model and treatment.

**
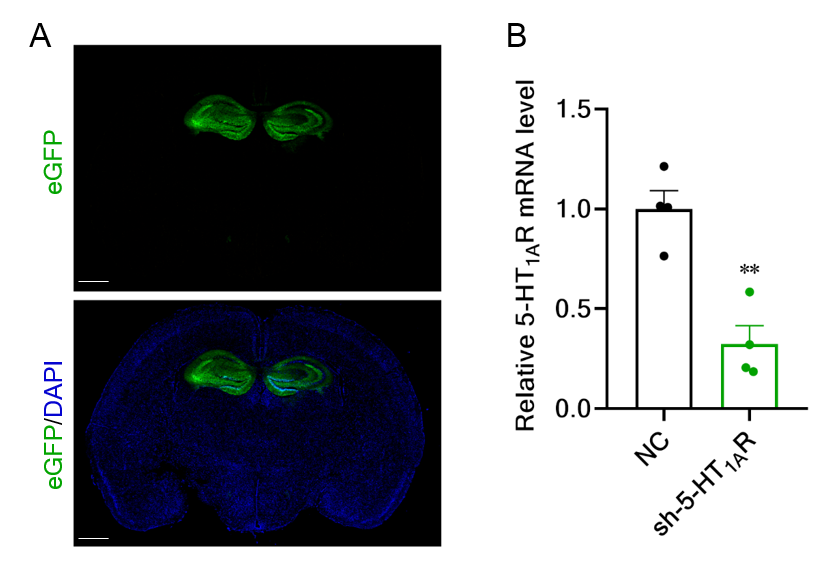
**

**Fig. S9 The efficiency of AAVs carrying sh-5HT_1A_R was validated in mice hippocampus.** A. Representative images of coronal slices of the mouse brain after administration of AAVs carrying eGFP-sh-5HT_1A_R. Scale bar: 1000 μm. B. Relative 5-HT_1A_R mRNA level in the hippocampus of mice after injection either with NC or sh-5HT_1A_R (n = 4). All the quantification data are presented as mean ± SEM. ^**^*P* < 0.01 versus NC group.

**
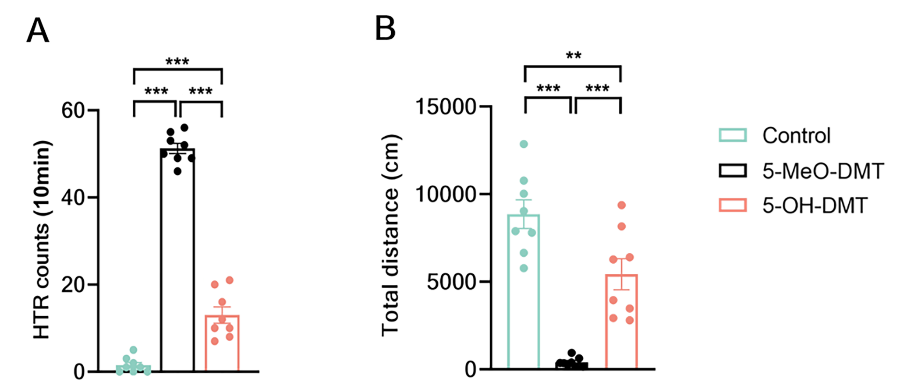
**

**Fig. S10 5-OH-DMT has fewer side effects than 5-MeO-DMT. A.** Number of head-twitch response (HTR), and **B.** Total distance after 5-MeO-DMT and 5-OH-DMT injection (n = 8). All the quantification data are presented as mean ± SEM. ^**^*P* < 0.01, ^***^*P* < 0.001.

Table S1. Cryo-EM data collection, refinement and validation statistics.

|  | | 5-MeO-DMT | 5-OH-DMT | 5-OH-TMT |
| --- | --- | --- | --- | --- |
| **Data collection and processing** |  | |  |  |
| Magnification | | 165000 | 165000 | 165000 |
| Voltage (kV) | | 300 | 300 | 300 |
| Electron exposure (e^–^/Å^2^) | | 50 | 50 | 50 |
| Defocus range (μm) | | -1.2 to -1.8 | -1.5 to -1.8 | -1.2 to -1.8 |
| Pixel size (Å) | | 0.73 | 0.73 | 0.73 |
| Symmetry imposed | | C1 | C1 | C1 |
| Final particle images (no.) | | 180,294 | 195,003 | 139,905 |
| **Map resolution (Å)**  FSC threshold | |  |  |  |
|  |  | 0.143 | 0.143 | 0.143 |
| Map resolution (Å) | | 2.63 | 2.54 | 2.59 |
| Map sharpening B factor (Å^2^) | | 92.7 | 82.9 | 83.4 |
| **Refinement** | |  |  |  |
| Initial model used (PDB code) | | Ab-Initio | Ab-Initio | Ab-Initio |
| Model resolution (Å)  FSC threshold | | 2.9 | 2.8 | 2.9 |
|  |  | 0.5 | 0.5 | 0.5 |
| Model-Map CC (mask) | | 0.62 | 0.58 | 0.59 |
| Model composition  Non-hydrogen atoms  Protein residues | |  |  |  |
|  |  | 8933 | 8992 | 8970 |
|  |  | 1125 | 1126 | 1125 |
| B factors (Å^2^)  Protein  Ligands  Waters | |  |  |  |
|  |  | 57.05 | 42.72 | 73.17 |
|  |  | 71.25  59.96 | 60.89  46.36 | 34.71  50.00 |
| R.m.s. deviations  Bond lengths (Å)  Bond angles (Å) | |  |  |  |
|  |  | 0.002 | 0.002 | 0.003 |
|  |  | 0.465 | 0.670 | 0.592 |
| Validation  MolProbity score  Clash score  Rotamer outliers (%) | |  |  |  |
|  |  | 1.31 | 1.32 | 1.55 |
|  |  | 4.57 | 5.00 | 8.52 |
|  |  | 0.73 | 0.42 | 0.21 |
| Ramachandran plot  Favored (%)  Allowed (%)  Disallowed (%) | |  |  |  |
|  |  | 97.56  2.35 | 97.74  2.26 | 97.56  2.44 |
|  |  | 0 | 0 | 0 |
|  |  |  |  |  |

Table S2. List of primers sequences for site-direct mutagenesis studies.

| **Receptor** | **primers** | **Forward** | **Reverse** |
| --- | --- | --- | --- |
| 5-MeO-DMT-5-HT1A | D116^3.32^A | GCCCTCGCCGTGCTGTGCTGCACCTCATCC | CAGCACGGCGAGGGCGATGAACAGGTCGCA |
|  | V117^3.33^F | CTCGACTTCCTGTGCTGCACCTCATCCATC | GCACAGGAAGTCGAGGGCGATGAACAGGTC |
|  | C120^3.36^A | CTGTGCGCGTGCACCTCATCCATCTTGCAC | GGTGCACGCGCACAGCACGTCGAGGGCGAT |
|  | T121^3.37^A | TGCTGCGCCTCATCCATCTTGCACCTGTGC | GGATGAGGCGCAGCACAGCACGTCGAGGGC |
|  | S199^5.42^A | ATCTATGCTACCTTTGGAGCTTTCTACATC | AAAGGTAGCATAGATAGTGTAGCCATGATC |
|  | T200^5.43^A | TATTCCGCTTTTGGAGCTTTCTACATCCCG | TCCAAAAGCGGAATAGATAGTGTAGCCATG |
|  | A203^5.46^F | TTTGGATTCTTCTACATCCCGCTGCTGCTC | GTAGAAGAATCCAAAGGTGGAATAGATAGT |
|  | W358 ^6.48^ A | CTCTGCGCCCTGCCCTTCTTCATCGTGGCT | GGGCAGGGCGCAGAGGATGAAGGTGCCCAT |
|  | F361^6.51^A | CTGCCCGCTTTCATCGTGGCTCTTGTTCTG | GATGAAAGCGGGCAGCCAGCAGAGGATGAA |
|  | F362^6.52^A | CCCTTCGCTATCGTGGCTCTTGTTCTGCCC | CACGATAGCGAAGGGCAGCCAGCAGAGGAT |
|  | A365^6.55^F | ATCGTGTTCCTTGTTCTGCCCTTCTGCGAG | AACAAGGAACACGATGAAGAAGGGCAGCCA |
|  | N386^7.39^A | ATAATCGCCTGGCTGGGCTACTCCAACTCT | CAGCCAGGCGATTATGGCGCCCAACAGGGT |
|  | G389^7.42^A | TGGCTGGCCTACTCCAACTCTCTGCTTAAC | GGAGTAGGCCAGCCAATTGATTATGGCGCC |
|  | Y390^7.43^A | CTGGGCGCCTCCAACTCTCTGCTTAACCCC | GTTGGAGGCGCCCAGCCAATTGATTATGGC |
| 5-OH-DMT-5-HT1A | I167^4.56^A | TTCCTCGCCTCTATCCCGCCCATGCTGGG | GATAGAGGCGAGGAAGCCAATAAGCCAAGT |
|  | I189^ECL2^A | TGCACCGCAAGCAAGGATCATGGCTACAC | CTTGCTTGCGGTGCATGCGTCGGGGTCCGA |
|  | D116^3.32^A | GCCCTCGCCGTGCTGTGCTGCACCTCATCC | CAGCACGGCGAGGGCGATGAACAGGTCGCA |
|  | V117^3.33^F | CTCGACTTCCTGTGCTGCACCTCATCCATC | GCACAGGAAGTCGAGGGCGATGAACAGGTC |
|  | C120^3.36^A | CTGTGCGCGTGCACCTCATCCATCTTGCAC | GGTGCACGCGCACAGCACGTCGAGGGCGAT |
|  | T121^3.37^A | TGCTGCGCCTCATCCATCTTGCACCTGTGC | GGATGAGGCGCAGCACAGCACGTCGAGGGC |
|  | S199^5.42^A | ATCTATGCTACCTTTGGAGCTTTCTACATC | AAAGGTAGCATAGATAGTGTAGCCATGATC |
|  | T200^5.43^A | TATTCCGCTTTTGGAGCTTTCTACATCCCG | TCCAAAAGCGGAATAGATAGTGTAGCCATG |
|  | A203^5.46^F | TTTGGATTCTTCTACATCCCGCTGCTGCTC | GTAGAAGAATCCAAAGGTGGAATAGATAGT |
|  | W358^6.48^A | CTCTGCGCCCTGCCCTTCTTCATCGTGGCT | GGGCAGGGCGCAGAGGATGAAGGTGCCCAT |
|  | F361^6.51^A | CTGCCCGCTTTCATCGTGGCTCTTGTTCTG | GATGAAAGCGGGCAGCCAGCAGAGGATGAA |
|  | F362^6.52^A | CCCTTCGCTATCGTGGCTCTTGTTCTGCCC | CACGATAGCGAAGGGCAGCCAGCAGAGGAT |
|  | A365^6.55^F | ATCGTGTTCCTTGTTCTGCCCTTCTGCGAG | AACAAGGAACACGATGAAGAAGGGCAGCCA |
|  | N386^7.39^A | ATAATCGCCTGGCTGGGCTACTCCAACTCT | CAGCCAGGCGATTATGGCGCCCAACAGGGT |
|  | G389^7.42^A | TGGCTGGCCTACTCCAACTCTCTGCTTAAC | GGAGTAGGCCAGCCAATTGATTATGGCGCC |
|  | Y390^7.43^A | CTGGGCGCCTCCAACTCTCTGCTTAACCCC | GTTGGAGGCGCCCAGCCAATTGATTATGGC |
| 5-OH-TMT-5-HT1A | I167^4.56^A | TTCCTCGCCTCTATCCCGCCCATGCTGGG | GATAGAGGCGAGGAAGCCAATAAGCCAAGT |
|  | D116^3.32^A | GCCCTCGCCGTGCTGTGCTGCACCTCATCC | CAGCACGGCGAGGGCGATGAACAGGTCGCA |
|  | V117^3.33^F | CTCGACTTCCTGTGCTGCACCTCATCCATC | GCACAGGAAGTCGAGGGCGATGAACAGGTC |
|  | C120^3.36^A | CTGTGCGCGTGCACCTCATCCATCTTGCAC | GGTGCACGCGCACAGCACGTCGAGGGCGAT |
|  | T121^3.37^A | TGCTGCGCCTCATCCATCTTGCACCTGTGC | GGATGAGGCGCAGCACAGCACGTCGAGGGC |
|  | S199^5.42^A | ATCTATGCTACCTTTGGAGCTTTCTACATC | AAAGGTAGCATAGATAGTGTAGCCATGATC |
|  | T200^5.43^A | TATTCCGCTTTTGGAGCTTTCTACATCCCG | TCCAAAAGCGGAATAGATAGTGTAGCCATG |
|  | A203^5.46^F | TTTGGATTCTTCTACATCCCGCTGCTGCTC | GTAGAAGAATCCAAAGGTGGAATAGATAGT |
|  | W358^6.48^A | CTCTGCGCCCTGCCCTTCTTCATCGTGGCT | GGGCAGGGCGCAGAGGATGAAGGTGCCCAT |
|  | F361^6.51^A | CTGCCCGCTTTCATCGTGGCTCTTGTTCTG | GATGAAAGCGGGCAGCCAGCAGAGGATGAA |
|  | F362^6.52^A | CCCTTCGCTATCGTGGCTCTTGTTCTGCCC | CACGATAGCGAAGGGCAGCCAGCAGAGGAT |
|  | A365^6.55^F | ATCGTGTTCCTTGTTCTGCCCTTCTGCGAG | AACAAGGAACACGATGAAGAAGGGCAGCCA |
|  | N386^7.39^A | ATAATCGCCTGGCTGGGCTACTCCAACTCT | CAGCCAGGCGATTATGGCGCCCAACAGGGT |
|  | G389^7.42^A | TGGCTGGCCTACTCCAACTCTCTGCTTAAC | GGAGTAGGCCAGCCAATTGATTATGGCGCC |
|  | Y390^7.43^A | CTGGGCGCCTCCAACTCTCTGCTTAACCCC | GTTGGAGGCGCCCAGCCAATTGATTATGGC |

Table S3. The treatment efficiency of 5-MeO-DMT and 5-OH-DMT in NC and sh-5-HT1AR mice with CUMS depression model.

| Efficiency | | | 5-MeO-DMT | | 5-OH-DMT | |
| --- | --- | --- | --- | --- | --- | --- |
| Group | | | NC | sh-5-HT_1A_R | NC | sh-5-HT_1A_R |
| Experiments | TST | | 80.21% | 77.37% | 83.06% | 68.76% |
|  | FST | | 31.03% | 37.07% | 54.46% | 46.50% |
|  | SPT | | 78.05% | 95.65% | 62.91% | 82.54% |
|  | MWM | Escape latency | 124.47% | 81.41% | 146.30% | 81.93% |
|  |  | Times across target | 76.47% | 110.00% | 111.76% | 110.00% |
|  | OFT | Total distance | 155.14% | 65.00% | 128.78% | 64.83% |
|  |  | Time in center | 75.13% | 56.37% | 148.54% | 86.35% |

The efficiency was calculated as: efficiency = (V_5-MeO-DMT or 5-OH-DMT_ - V_CUMS_) / (V_Control_ - V_CUMS_); V, Value.
