# Supplementary figures and images for: Structural Pharmacology of Bufotenine Derivatives in Activating the 5-HT1A Receptor for Therapeutic Potential in Depression and Anxiety
Source: Research (Wash D C). 2025 Dec 23;8:0987. doi: 10.34133/research.0987 (PMC12722636; doi:10.34133/research.0987)

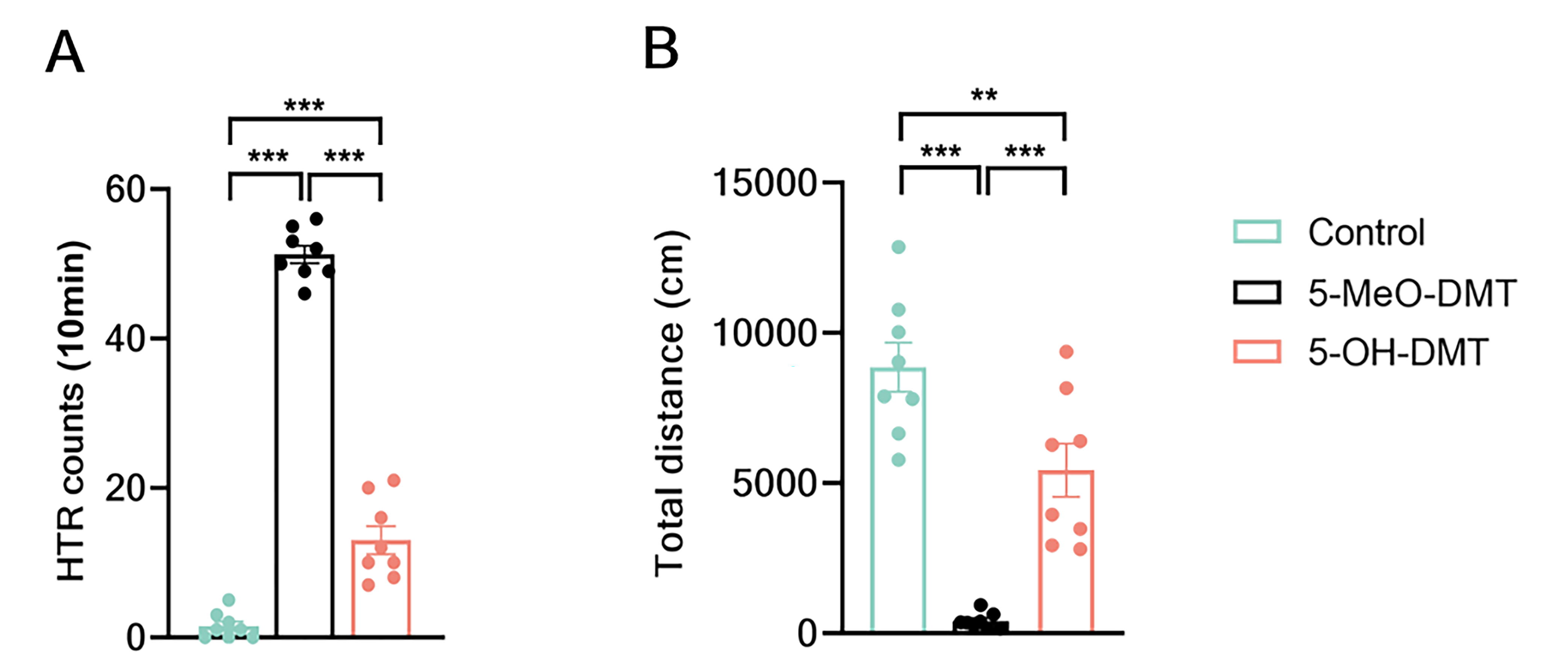

Supplement: Supplementary 1 — Figs. S1 to S10 Tables S1 to S3 [file research.0987.f1.zip › Fig. S10.tif]

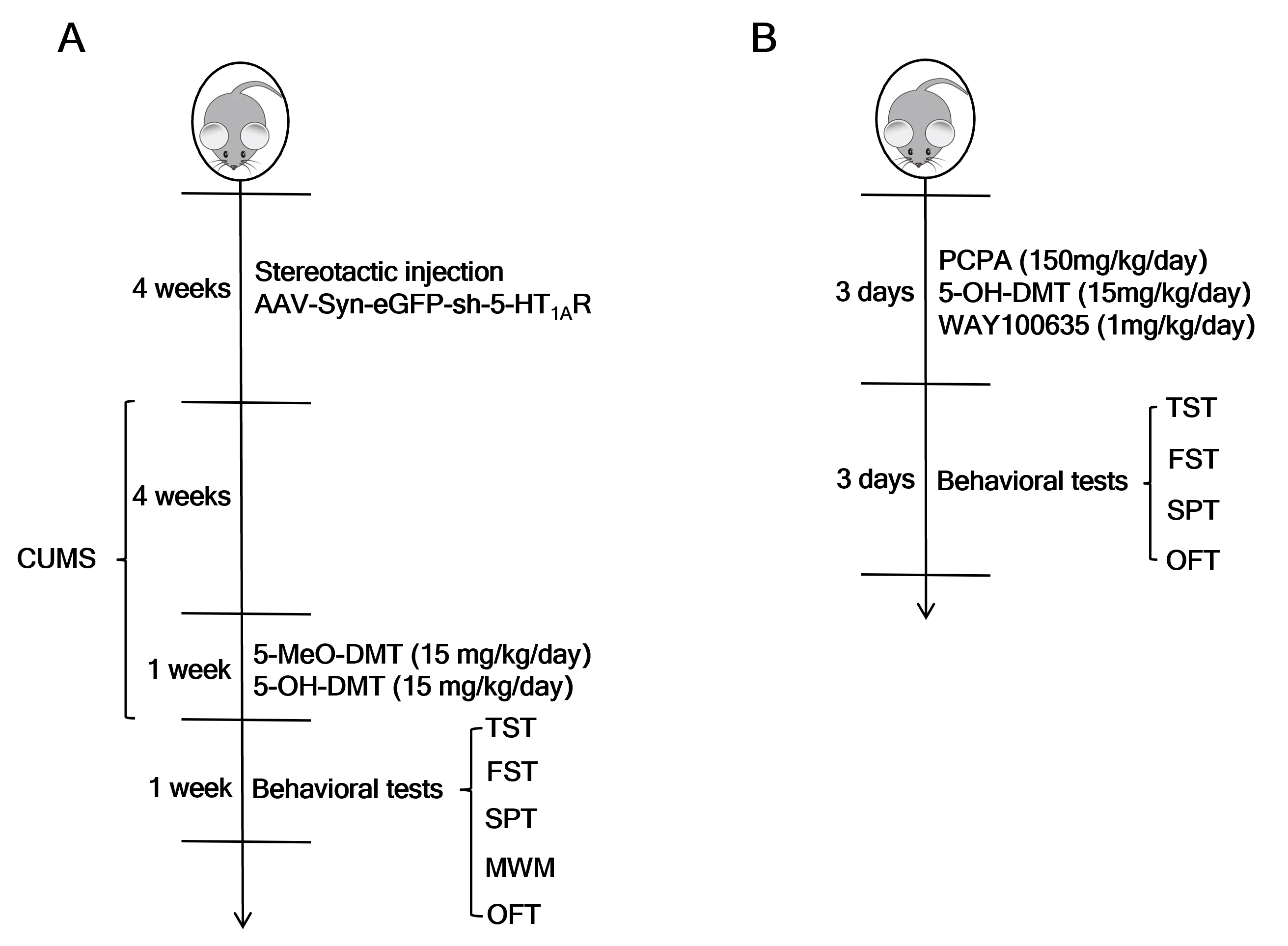

Supplement: Supplementary 1 — Figs. S1 to S10 Tables S1 to S3 [file research.0987.f1.zip › Fig. S8.tif]

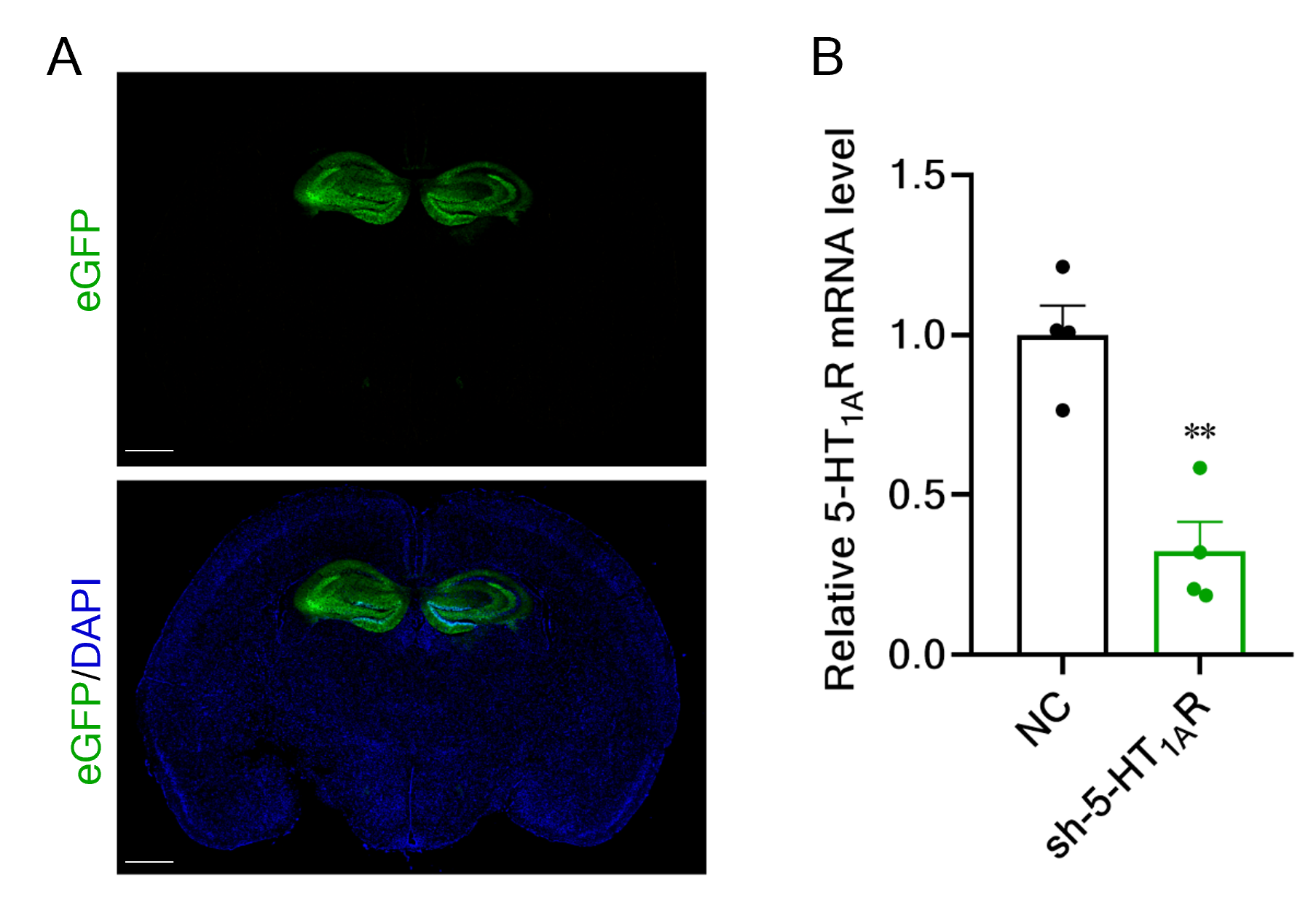

Supplement: Supplementary 1 — Figs. S1 to S10 Tables S1 to S3 [file research.0987.f1.zip › Fig. S9.tif]

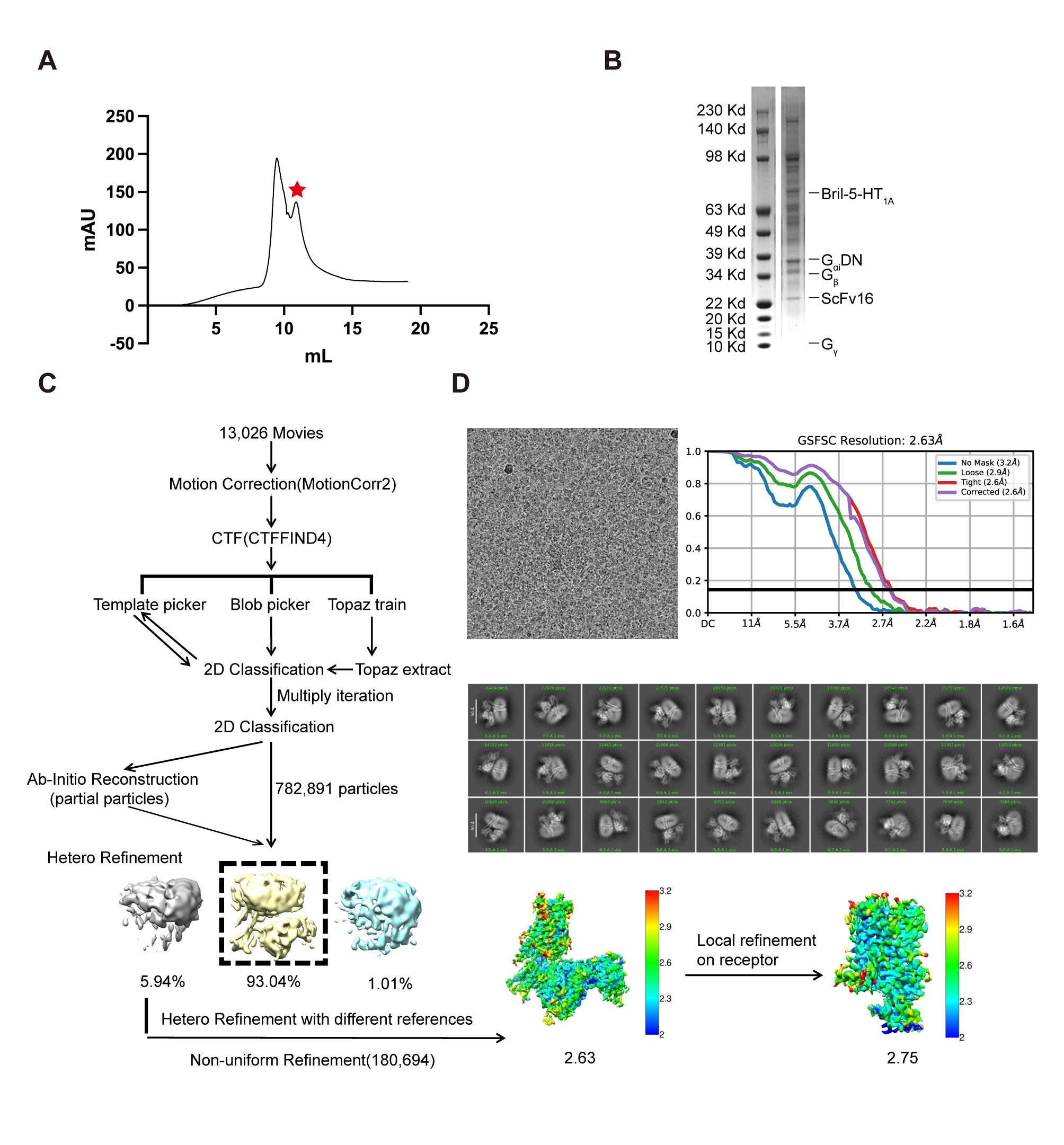

Supplement: Supplementary 1 — Figs. S1 to S10 Tables S1 to S3 [file research.0987.f1.zip › S Figure1 .tif]

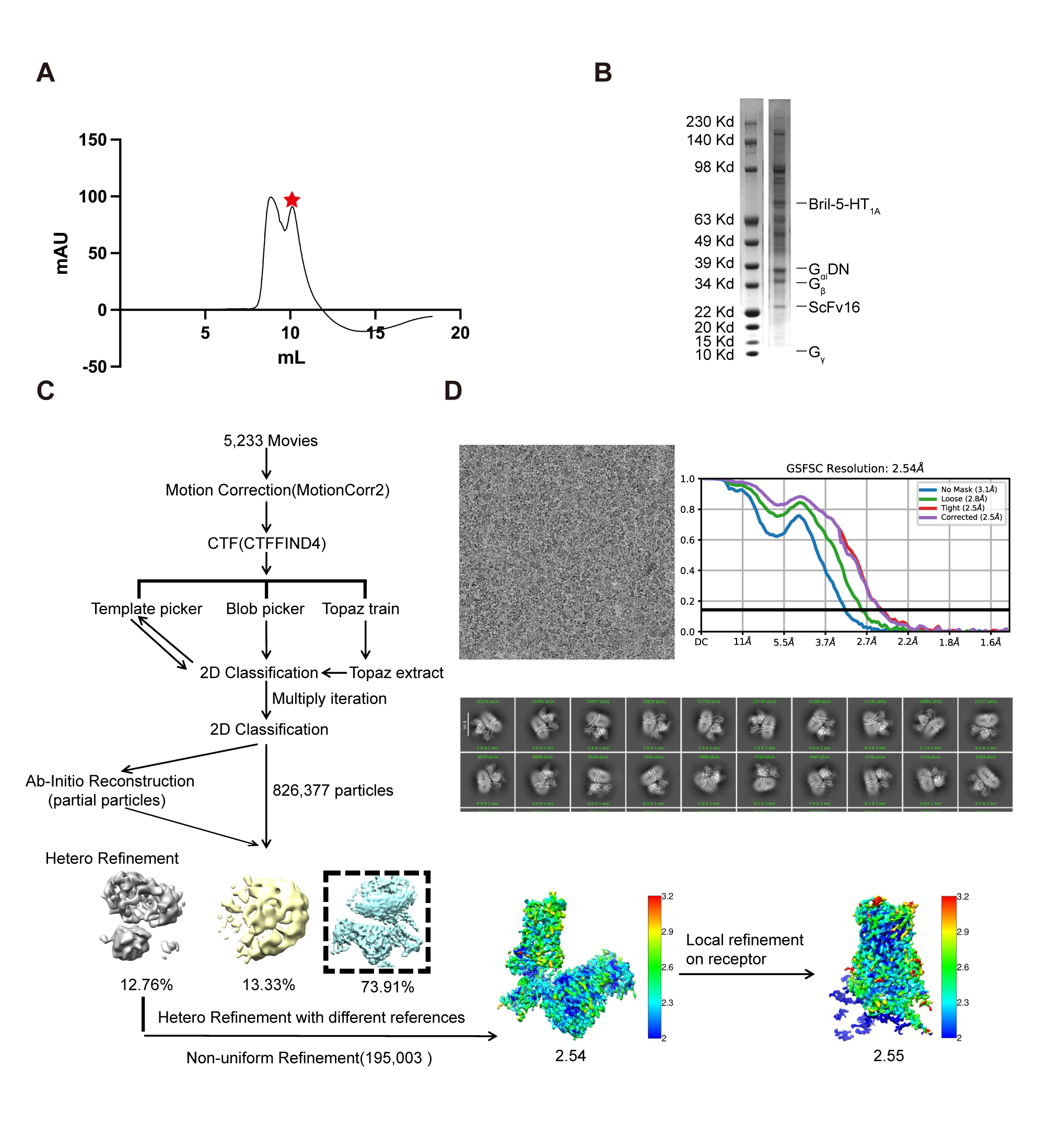

Supplement: Supplementary 1 — Figs. S1 to S10 Tables S1 to S3 [file research.0987.f1.zip › S figure2.tif]

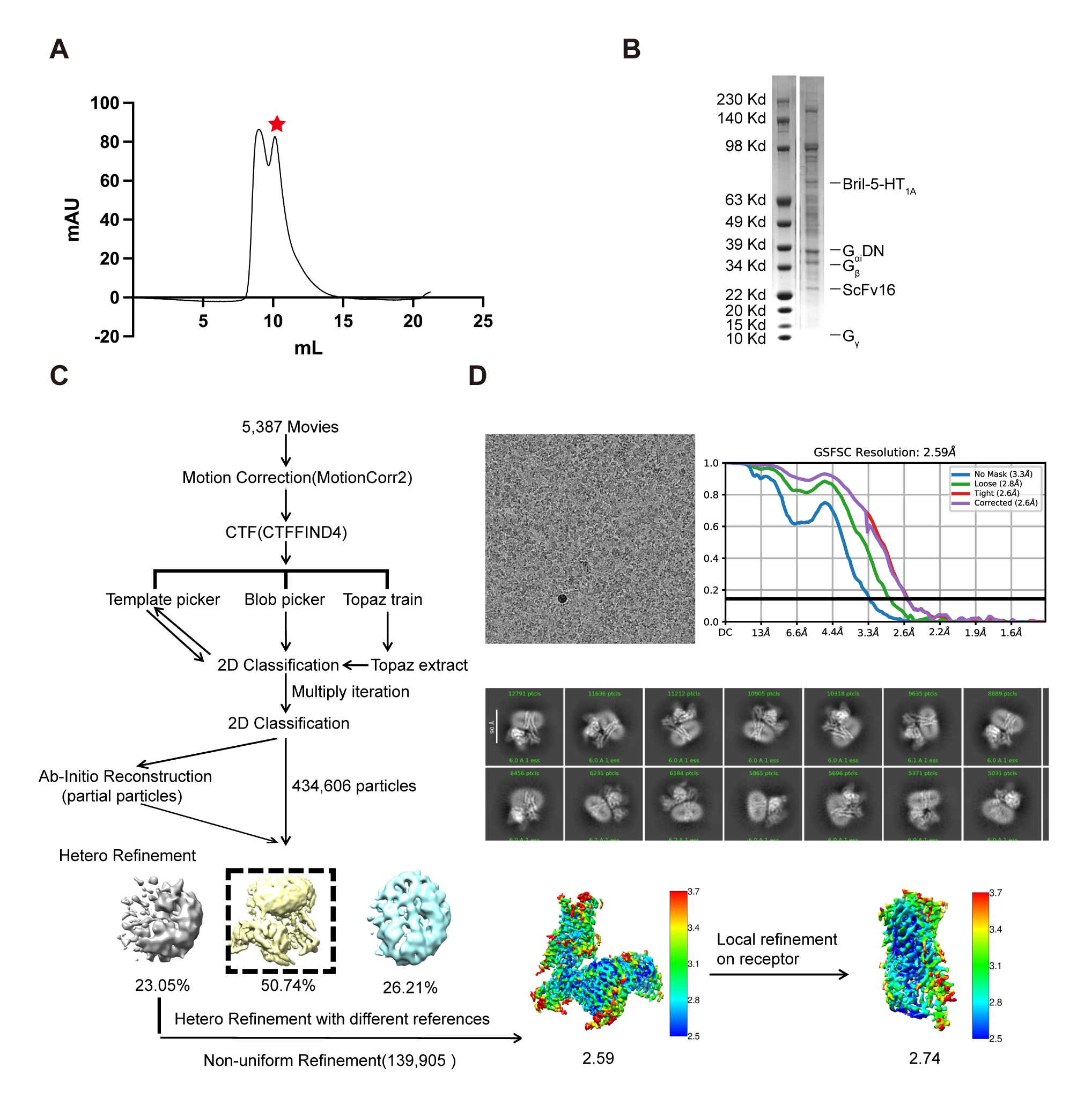

Supplement: Supplementary 1 — Figs. S1 to S10 Tables S1 to S3 [file research.0987.f1.zip › S figure3.tif]

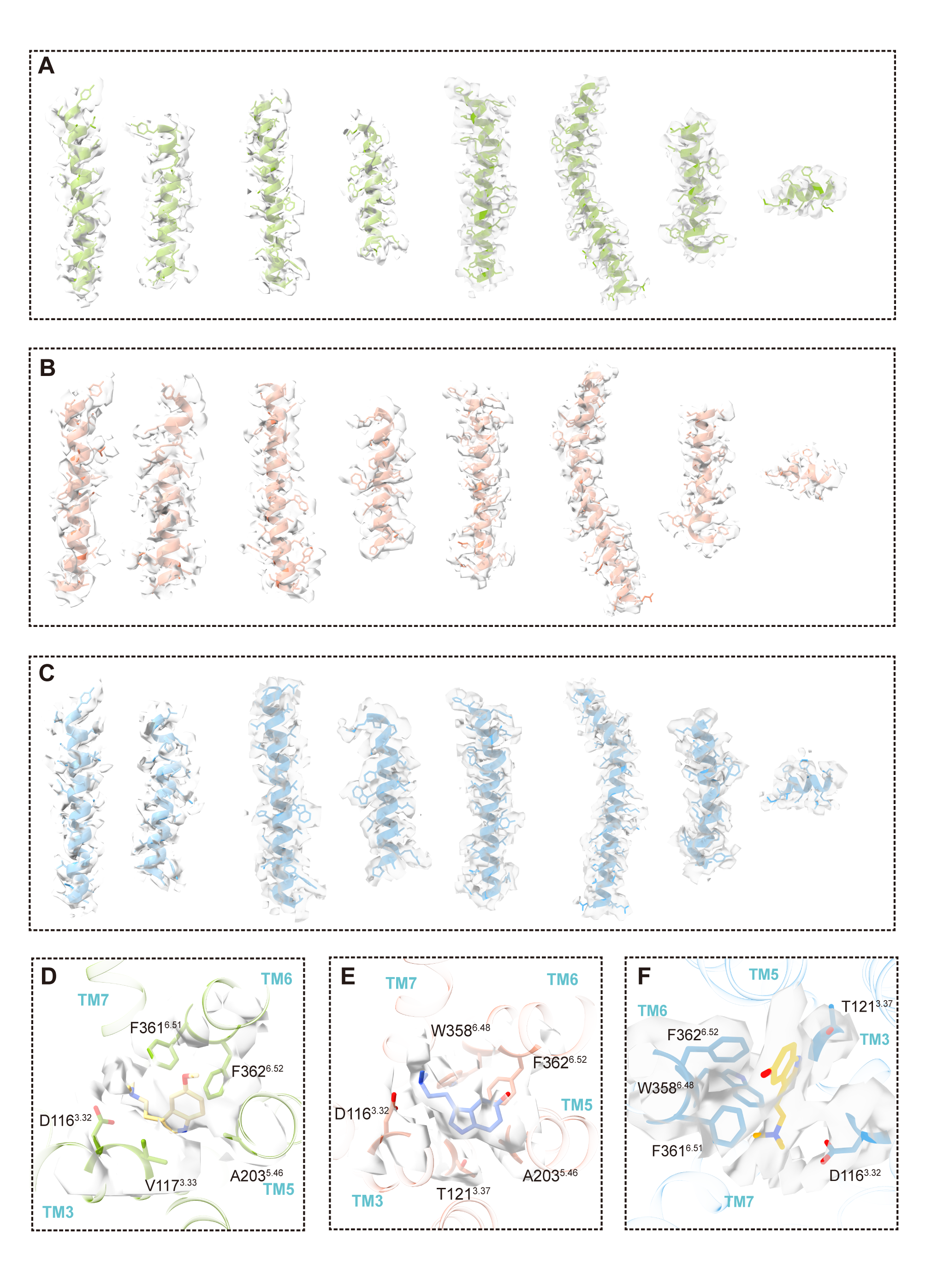

Supplement: Supplementary 1 — Figs. S1 to S10 Tables S1 to S3 [file research.0987.f1.zip › S figure4.tif]

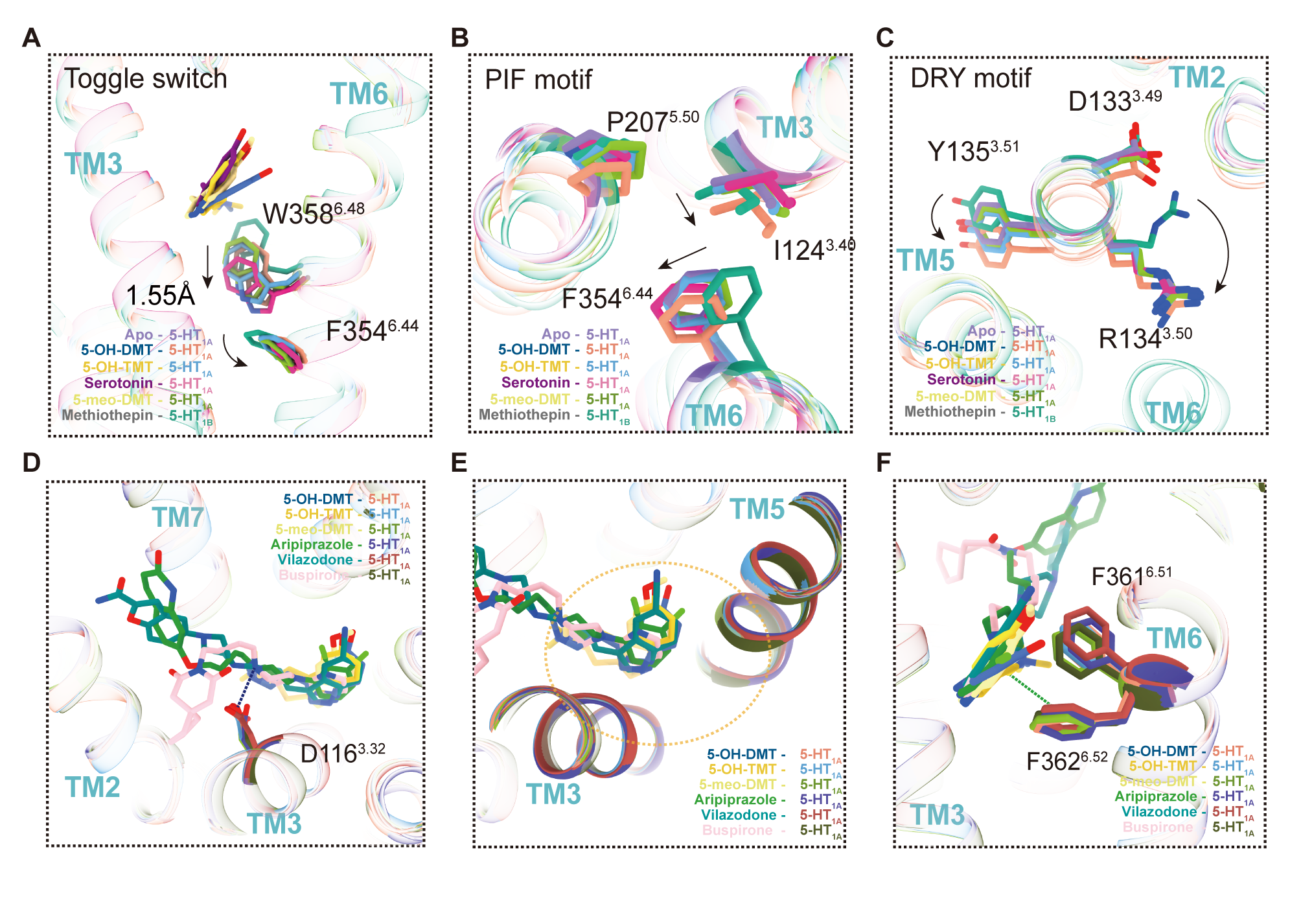

Supplement: Supplementary 1 — Figs. S1 to S10 Tables S1 to S3 [file research.0987.f1.zip › S Figure5 20250915.tif]

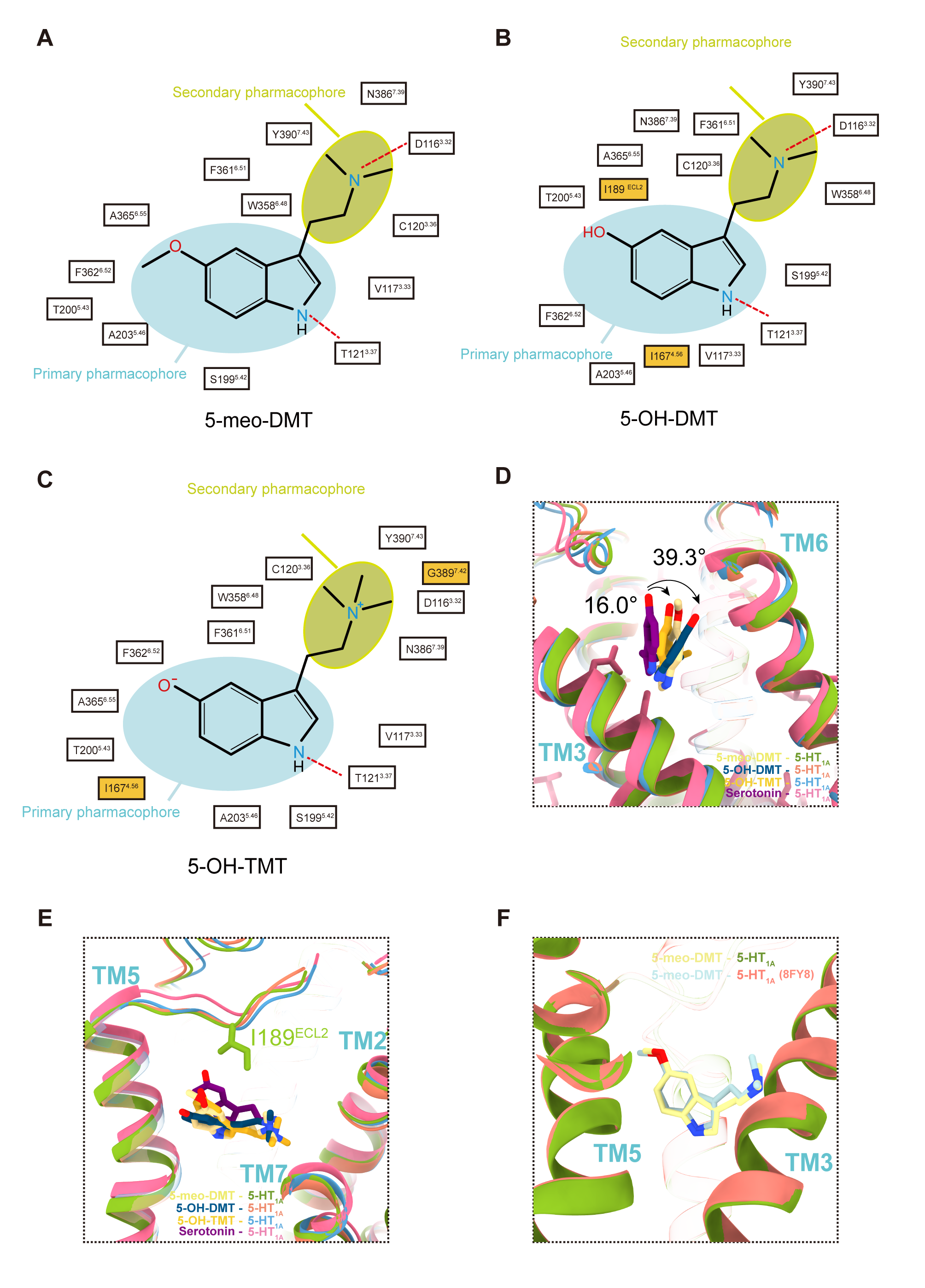

Supplement: Supplementary 1 — Figs. S1 to S10 Tables S1 to S3 [file research.0987.f1.zip › S Figure6 20250921.tif]

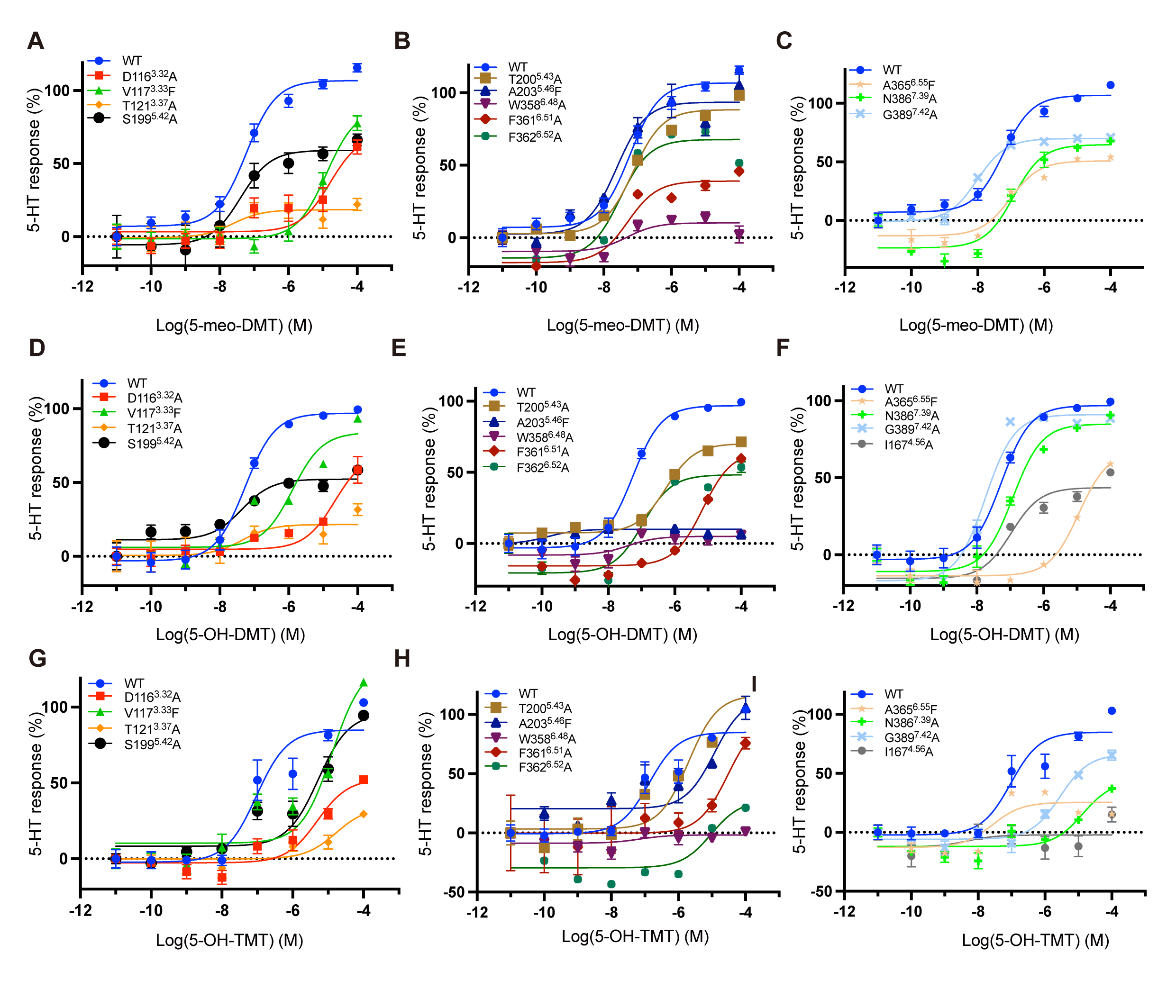

Supplement: Supplementary 1 — Figs. S1 to S10 Tables S1 to S3 [file research.0987.f1.zip › S Figure7.tif]
